# A Portable and Dual‐Button Microneedle Device Enables Intelligent Multimodal Laser Sensing

**DOI:** 10.1002/advs.75564

**Published:** 2026-05-07

**Authors:** Yuanchao Liu, Xiujuan Hu, Shengqun Shi, Bowen Li, Zhixing Ge, Yunchen Long, Chaochao Sun, Annan Chen, Bingbing Gao, Lianbo Guo, Condon Lau, Wei Luo, Chwee Teck Lim

**Affiliations:** ^1^ Department of Physics City University of Hong Kong Kowloon Hong Kong SAR China; ^2^ Department of Biomedical Engineering National University of Singapore Singapore Singapore; ^3^ School of Integrated Circuits Huazhong University of Science and Technology Wuhan China; ^4^ Wuhan National Laboratory for Optoelectronics (WNLO) Huazhong University of Science and Technology Wuhan China; ^5^ Department of Mechanical Engineering City University of Hong Kong Kowloon Hong Kong SAR China; ^6^ Department of Materials Science and Engineering City University of Hong Kong Kowloon Hong Kong SAR China; ^7^ School of Pharmaceutical Sciences Nanjing Tech University Nanjing China; ^8^ College of Information and Intelligence Engineering Zhejiang Wanli University Zhejiang China; ^9^ Institute For Health Innovation and Technology National University of Singapore Singapore Singapore

**Keywords:** 3D printing, artificial intelligence, microfluidics, microneedles, surface‐enhanced Raman spectroscopy

## Abstract

Frequent blood testing remains invasive and impractical, hindering real‐time health monitoring. Interstitial fluid (ISF) offers a promising alternative, yet current microneedle platforms are complex, inefficient, and lack multiplexing capability. Here, we present a portable and dual‐button microneedle device for rapid ISF sampling, coupled with multimodal laser sensing for molecular and elemental analysis. The disposable device has a low material cost (< 2 USD). The dual‐button design ensures user‐friendly microneedle operation, while the hollow microneedles with built‐in microfluidic channels enable ISF sampling within 1 min. In parallel, gold nanocubes coupled with MXene are engineered as a sensing module, providing signal enhancement for laser spectroscopy in molecular and elemental analysis. Furthermore, artificial intelligence (AI)‐assisted data processing enhances spectral data interpretation for comprehensive health assessment. Both in vitro and in vivo studies demonstrate promising performance (with accuracies exceeding 88%). This user‐friendly, rapid, cost‐effective, and multiplexed device offers a powerful route toward clinical translation, bridging the gap between advanced laser spectroscopy and point‐of‐care applications.

## Graphical abstract

A portable and dual‐button microneedle device enables rapid interstitial fluid sampling. Coupled with multimodal laser sensing and AI‐assisted data processing, the platform enables simultaneous molecular and elemental analysis for minimally invasive and multiplexed health assessment toward point‐of‐care diagnostics.

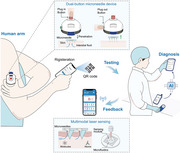

## 1 Introduction

As the global burden of chronic and infectious diseases continues to rise, there is a pressing demand for diagnostic strategies that are rapid, minimally invasive, and cost‐effective [1, 2, 3]. While blood analysis remains the clinical gold standard, it's constrained by infection risks, patient discomfort, and procedural complexity [4]. In contrast, interstitial fluid (ISF) offers a promising alternative due to its minimally invasive nature and its rich content of metabolites, providing low‐risk, less‐pain, and high‐accuracy [5, 6, 7]. Despite this potential of ISF‐based diagnostics, current ISF sampling techniques, such as suction blisters and microdialysis [8], are limited by high costs, complexity, and inefficiency, posing a major barrier to widespread clinical adoption.

Microneedles have recently attracted attention for their minimally invasive, cost‐effective, and user‐friendly properties in ISF collection [9, 10, 11]. These microneedle tips can penetrate the epidermis without reaching deeper blood vessels or nerves, thereby enabling rapid ISF extraction with minimal discomfort. Beyond sampling, other biomedical functions have been explored, for example, drug delivery, and therapy [12, 13, 14]. Moreover, microneedles can be integrated with diverse sensing modalities, such as electrochemical [15, 16] and optical sensors [17], to enhance detection capabilities. For example, Wang et al. [18] developed microneedles integrated with reusable electronics for wireless and continuous real‐time sensing of two metabolites with high accuracy and sensitivity. Sang et al. [19] proposed a fluorescence‐based biodegradable microneedle sensor array for tether‐free continuous glucose monitoring using a smartphone application. However, the chemical complexity of ISF poses challenges for electrochemical sensing, including electrode fouling, cross‐sensitivity, and limited calibration stability, while multiplexed detection often requires more complex electrode integration. Similarly, although fluorescence‐based sensors can support multiplexed detection through multicolor labeling, their application to simultaneous multi‐analyte ISF analysis remains limited by spectral overlap and labeling complexity [20, 21].

In contrast, multimodal laser sensing techniques, such as surface‐enhanced Raman spectroscopy (SERS) and laser‐induced breakdown spectroscopy (LIBS), provide label‐free detection with high molecular specificity, minimal signal interference, and intrinsic capability for multiplexed biomarker analysis [22, 23]. Because ISF contains biomarkers at both the molecular and elemental levels, relying on a single sensing modality may provide only partial information. Specifically, SERS provides molecular‐level fingerprinting through vibrational signatures [24, 25], while LIBS provides rapid elemental analysis [22]. Integrating these two techniques therefore enables more comprehensive ISF characterization than either modality alone, making this strategy attractive for multi‐biomarker analysis.

To date, several microneedle‐based SERS systems have been reported, but the integration of LIBS into microneedle‐enabled ISF analysis remains largely unexplored. Generally, hydrogel extraction [26] and microneedle functionalization [27, 28] are used to obtain sufficient analytes and enhance diagnostic capability. Meanwhile, most studies have focused on technological advancements of microneedles while performances in use still remain innovation, such as ease of use, patient acceptance, and prevention of contamination during collection. Therefore, effectively addressing these factors is critical for translating microneedle‐based diagnostics into clinical practice.

To overcome these challenges, we developed a portable dual‐button microneedle device that integrates rapid ISF extraction, multimodal spectral sensing, and AI‐assisted analysis for comprehensive health monitoring. The device features 3D‐printed hollow microneedles and a user‐friendly dual‐button applicator for rapid ISF extraction. This dual‐button is designed to reduce the risk of contamination and cross‐infection by preventing direct contact or misuse. The plasmonic sensing module (gold nanocubes (Au NCs) coupled with MXene) is applied for multi‐modal spectral detection to obtain molecular and elemental information, followed by artificial intelligence (AI)‐assisted multimodal spectral fusion and classification. Experimental tests confirmed the device's accuracy and stability, while animal trials demonstrated its practical potential, suggesting a promising path toward clinical application. In summary, this low‐cost and user‐friendly device represents a practical advancement for point‐of‐care testing (POCT), paving the way for broader clinical adoption and improved patient outcomes.

## 2 Materials and Methods

### 2.1 Materials and Reagents

Two types of 3D printing resin (BioMed Clear Resin for the microneedles and Tough 2000 resin for the applicator) were acquired from Formlabs (USA). The MXene (single‐layer Ti_3_C_2_T_X_, 5 mg/mL) was brought from Xinxi Tech. Co., Ltd. (Guangdong, China). The Au NCs (50 nm in diameter, 0.5 mm) were brought from Beijing Zhongke Keyou Technology Co., Ltd. (China). Filter papers were purchased from BKMAM Biotech (China). Moreover, agarose (BioReagent grade), methylene blue (MB, ≥82%), 6‐mercaptopurine monohydrate (6‐MP, 98%), lithium carbonate (Li_2_CO_3_, ACS reagent, ≥99.0%), manganese sulfates (MnSO_4_, ACS reagent, ≥98%), glucose (≥99.5%), and uric acid (≥99%) were all purchased from Sigma Aldrich (USA).

### 2.2 Equipment

The stereolithography (SLA) 3D printer was purchased from Formlabs (Form 3B, USA). The Nikon D7100 digital camera, equipped with a 105 mm f/2.8 lens, was used for taking photos. The scanning electron microscope (SEM, Quanta 200, FEI, USA) was used for morphological characterization. The UV–vis spectrophotometer (Lambda 35, PerkinElmer, USA, 400–800 nm) was used to record the absorption spectra of Au NCs and MXene solutions. In vitro skin testing was conducted using the VivoSight DX optical coherence tomography (OCT) system. Then, X‐ray diffraction (XRD, Rigaku, Japan) was performed to analyze crystalline structures. Inductively coupled plasma mass spectrometry (ICP‐MS, PerkinElmer NexION 300X) was performed for element detection. The liquid chromatography‐mass spectrometry (LC‐MS, Ultimate 3000 UHPLC‐Q Exactive) was employed to analyze molecular compositions.

A confocal Raman spectrometer (Horiba XploRA, 638 nm wavelength) was used for SERS detection. The LIBS system was developed in our group [29, 30]. Specifically, an Nd:YAG laser (Nimma 400, Beamtech Optronics, China) produces a pulsed laser beam (532 nm, 50 mJ), which is directed through an optical structure to ablate the sample surface. The emitted light from the plasma was captured and processed by a spectrometer (AvaSpec‐EVO, Avantes B.V., Netherlands) coupled with a complementary metal‐oxide‐semiconductor (CMOS) detector. A digital delay generator (LDG3.0, Wuhan N&D Laser, China) was used to synchronize the operation of the laser and spectrometer. Subsequently, the sample spectra were obtained for analysis.

### 2.3 Device Preparation

The applicator was designed in SolidWorks and printed using the SLA 3D printer (Form 3B, Formlabs) with Tough 2000 resin. After printing, the parts were cleaned and subjected to secondary UV curing to enhance their mechanical strength. The components were then assembled, including the integration of custom‐designed springs for the two buttons. Finally, various parts of the applicator were painted with Tamiya dyes in colors to enhance product identification and user acceptance.

The microneedle was initially designed using AutoCAD and fabricated with the same 3D printer using BioMed Clear Resin. After printing, the microneedle underwent post‐processing, including being removed from the printer bed and ultrasonic cleaning in isopropanol (IPA) for 30 min. Afterward, the microneedle was UV‐cured at 60°C for 30 min. After UV curing, the support structures were removed to complete the fabrication process.

### 2.4 Plasmonic Substrate Preparation

The SERS substrate was fabricated via a low‐cost, straightforward, and rapid process. Briefly, 5 mL of MXene solution was mixed with 5 mL of Au NCs solution and ultrasonicated to ensure uniform dispersion. The resulting mixture (10 mL) was then vacuum‐filtered onto filter paper. After filtration, the filter paper was dried and subsequently punched into discs (7 mm in diameter) to match the collection area on the back of the microneedle device.

### 2.5 Artificial Skin Preparation

Artificial skin was prepared using agarose gel as the base material. A 2% (w/v) agarose solution was prepared by dissolving agarose powder in deionized water, which was then heated with stirring until completely dissolved. After complete dissolution, the solution was cooled and poured into a petri dish for solidification at room temperature, in which the agarose gel formed a stable matrix mimicking the structure and properties of human skin. Separately, MB solution (1 mm) was added to the agarose solution at a 1:10 volume ratio. The mixture was stirred until homogeneous and then poured into a mold. After solidification, the agarose–MB gels formed a stable skin model for experimental use.

### 2.6 In Vitro Testing

#### 2.6.1 Mechanical Properties Characterization

The mechanical properties of the microneedle tips were evaluated using a mechanical testing system (CellScale, Canada) under varying pressures. The penetration performance of the device was also assessed by recording the corresponding mechanical data during its insertion into artificial skin.

#### 2.6.2 Biocompatibility Testing

The biocompatibility of the microneedle tips was assessed using human dermal fibroblasts (HDFs). The microneedles were co‐cultured with HDFs for 1, 3, and 5 days. The cells were then incubated with 1–5 µm Calcein‐AM and 0.5–1 µg/mL PI solutions for 20–30 min at room temperature. Live cells exhibited green fluorescence (Calcein‐AM), while dead cells showed red fluorescence (PI), observed under a fluorescence microscope. The results of cell viability were used to assess any cytotoxic effects of the microneedle tips, thereby verifying their safety for both in vitro and in vivo applications.

#### 2.6.3 Antibacterial Testing

The antibacterial properties of the sensing module were evaluated. The sensing module was co‐cultured with E. coli (10^5^ CFU/mL) at 37°C for 24 h. After incubation, the bacterial suspension was eluted with PBS and diluted 10‐fold and 10^2^‐fold. The original and diluted suspensions were evenly spread onto LB agar plates and incubated at 37°C for 18 h. The plates were then removed, photographed, and the colony counts were recorded for quantitative analysis.

### 2.7 In Vivo Testing

In vivo experiments were performed using Sprague‐Dawley (SD) rats. Initially, the skin penetration test was performed by inserting microneedles into the dorsal skin of the rats. Then, the skin at the penetration site was sectioned into 2 µm slices and stained with hematoxylin and eosin (H&E). The stained slices were imaged using an inverted microscope (IX83, Olympus) to evaluate the penetration depth. Moreover, the recovery process of the dorsal skin, after microneedle penetration, was monitored at regular intervals to assess tissue recovery. Meanwhile, the OCT was employed to monitor the microneedles insertion process, skin recover process, and microneedles’ dissolution within the skin.

### 2.8 Data Analysis

Solutions containing various atomic or molecular biomarkers were injected into the artificial skin to simulate ISF, and then extracted using microneedles. After extraction, the biomarkers were analyzed by multi‐modal laser sensing (SERS and LIBS), enabling assessment of atomic and molecular analytes. Specifically, SERS and LIBS were sequentially performed within the same sensing region. SERS was conducted prior to LIBS to avoid interference from LIBS‐induced ablation. Data analysis of the collected spectra was then performed by indicators such as coefficient of determination (R^2^), standard deviation (SD), relative standard deviation (RSD), and limit of detection (LOD).

To enable accurate spectral data interpretation, multiple data fusion and deep learning algorithms were employed. A convolutional neural network (CNN) was used to extract and classify features from the fused SERS and LIBS spectra [31, 32]. The uniform manifold approximation and projection (UMAP) algorithm was applied for 2D visualization of high‐dimensional spectral features, facilitating the intuitive assessment of class separation [33]. Additionally, receiver operating characteristic (ROC) curves were generated, and the corresponding area under the curve (AUC) values were used to evaluate model classification performance.

### 2.9 Animal Experiments

In this study, a total of eighteen mice (C57BL/6) were used and divided into three groups (1, 2, and 3). All animal experiments were reviewed and approved by the Institutional Animal Care and Use Committee (IACUC) of Ruige Biotechnology with ethical approval number: 20240923‐001. Each group consisted of six mice with an equal distribution of males and females. The mice were maintained under the same standard conditions until adulthood. Prior to drug injection and sampling, all the mice were fasted for 24 h, with free access to water, to minimize the potential influence of food intake on the outcomes.

Groups 1 and 2 were designated as experimental groups and received 200 µL of 6‐MP (2 mg/mL) and Li (2 mg/mL), respectively, via tail vein injection. The third group served as a control group and did not receive any treatment. Sampling was conducted at 2 and 4 h post‐injection. ISF was extracted from the dorsal skin using microneedles, while blood samples were collected from the facial vein using syringes. The blood was centrifuged at 3000 rpm for 10 min to separate plasma, which was then stored at −80°C. All procedures were executed by trained personnel to ensure precision and good animal welfare practice.

Finally, the ISF samples underwent SERS and LIBS analysis to detect 6‐MP and Li, respectively. The plasma samples were analyzed using LC‐MS and ICP‐MS to detect 6‐MP and Li, respectively, evaluating the reliability and accuracy of the above spectral results.

## 3 Results and Discussion

### 3.1 Device Concept, Workflow, and Functional Mechanism

**FIGURE 1:**
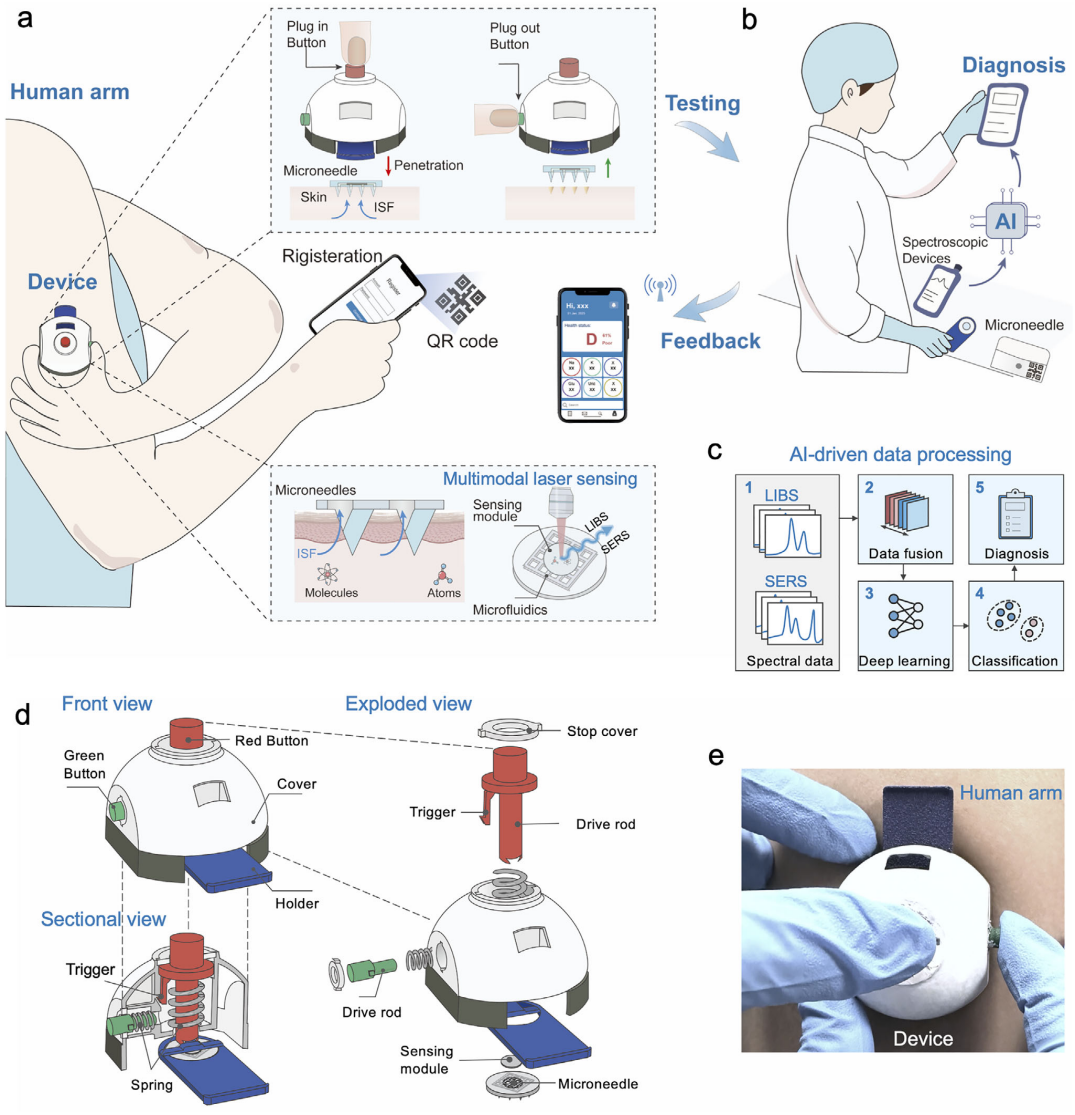
A portable and dual‐button device for rapid interstitial fluid (ISF) collection and multi‐modal laser sensing. (a,b) Schematic illustrations of the device's work process, including ISF sampling, testing, diagnosis, and feedback. (c) Schematic showing artificial intelligence (AI)‐driven data processing. (d) Schematic illustration of front view, sectional view, and exploded view of the device. (e) Photograph of the device on a human arm.

Reliable access to ISF remains a major bottleneck for clinical translation, as current sampling methods are complex and often cause discomfort. To address this, we developed a dual‐button microneedle device that simplifies ISF collection and integrates multimodal spectroscopy with AI‐assisted analysis. Figure 1a,b outlines the operational workflow, covering the entire process from ISF collection to diagnostic feedback. Users begin by scanning a QR code to register their contact information for diagnostic feedback. The device operates in two simple steps. In the first step, pressing the red button inserts the microneedles into the skin for ISF collection via a microfluidics channel. After about 1 min, pressing the green button retracts the microneedles. Once ISF is collected, the device is sent to the testing center. Here, multimodal laser sensing, including SERS and LIBS, is conducted on the sensing module, providing molecular and elemental information from ISF. After spectral acquisition at the testing center, the data are uploaded to the cloud for AI‐driven data processing (Figure 1c, multi‐spectral data fusion and deep learning), and diagnostic feedback is sent to users via mobile application or email. The AI is implemented as a companion digital health platform rather than being embedded in the device itself. Figure 1d shows the front, exploded, and sectional views of the device. The front view displays the complete appearance, featuring a white cover, a holder, and two buttons (a red button for microneedle insertion and a green button for microneedle release). The exploded view details the individual components, including the stop cover, trigger, spring, drive rod, and other key parts, clarifying their interconnections. In detail, the trigger locks and releases the mechanism, while the spring stores elastic energy to drive microneedle retraction. The drive rod transmits motion from the button to the microneedles, enabling insertion and retraction. The holder fixes and protects the microneedle array, preventing direct contact by users’ hands and thus reducing contamination risk. The sectional view (Figure 1d) illustrates the internal assembly of the device, demonstrating the applicator's operating mechanism and driving system. Together, these views offer a comprehensive visualization of the device's design, facilitating a deeper understanding of its working principles. A photograph of the device in use is shown in Figure 1e, with the sampling site positioned on the patient's shoulder.

**FIGURE 2:**
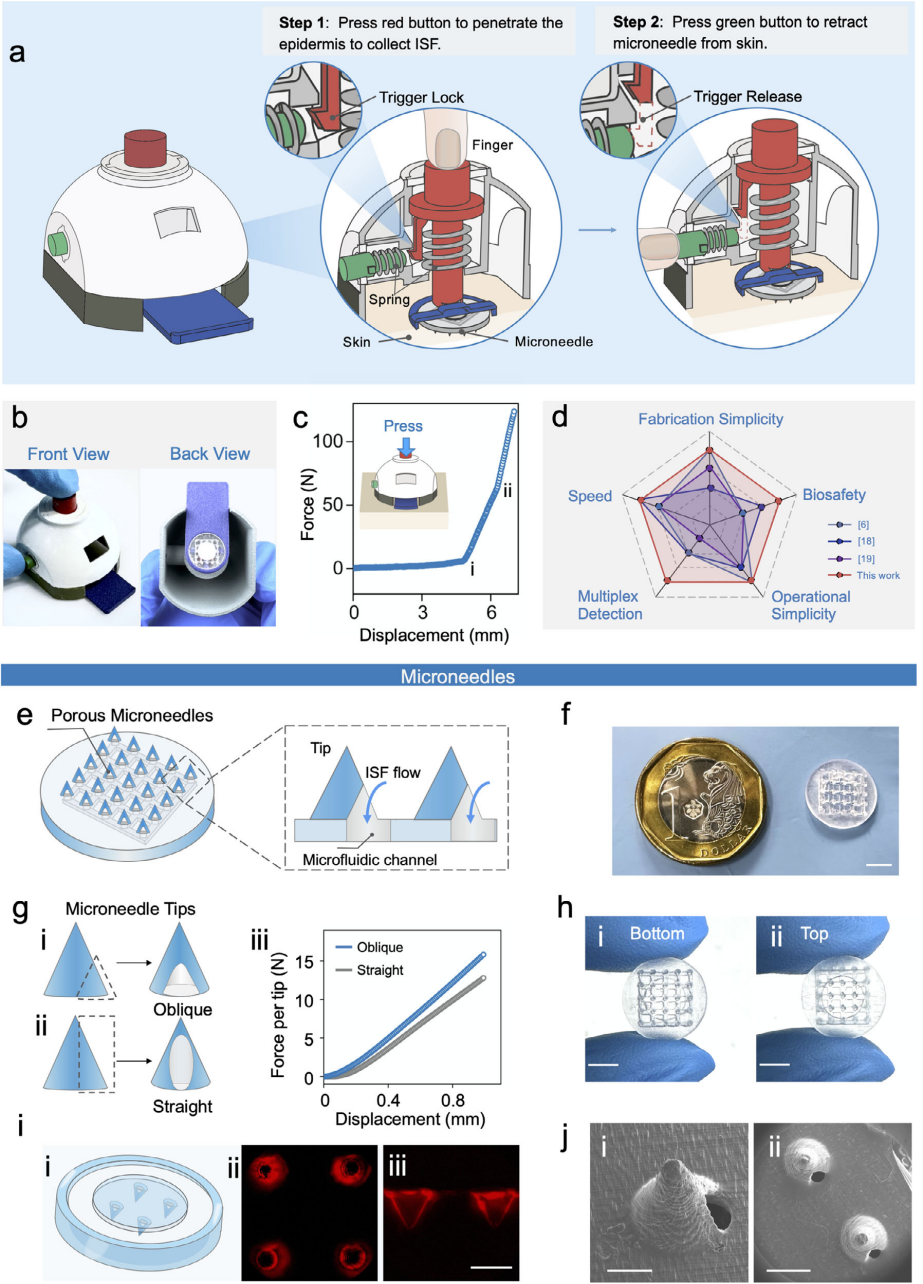
Characterization of the device's design of mechanical structure and microneedles for rapid ISF collection. (a) Sectional view schematic showing work process of the dual‐button system. (b) Photograph of the front view and back view of the fully integrated device. (c) The force‐displacement curve of the device penetrates into the artificial skin. (d) Radar chart comparing the performance with previously reported devices. (e) Schematic illustration of the porous microneedle tips. (f) Photography of a microneedle with a 10 Singapore dollar coin. Scale bar: 5 mm. (g) Comparison of two microneedle tips with different channel. (h) Photograph of a microneedle with a bottom view and a top view. Scale bar: 5 mm. (i) Fluorescence microscopy image showing microneedle penetration into artificial skin. Scale bar: 1 mm. (j) SEM images of microneedle tips. Scale bar: 0.5 mm for i and 1 mm for ii.

Beyond workflow, the device employs a dual‐button mechanical system to ensure reproducible and easy‐to‐use microneedle insertion and retraction (Figure 2a). Mechanically, this two‐step process operates as follows: pressing the red button initiates the downward movement of the drive rod, which simultaneously drives the microneedles to penetrate the skin for ISF collection and locks the trigger to maintain sampling. After ISF collection, pressing the green button releases the locked trigger, allowing the spring‐loaded drive rod to retract the microneedles and complete the sampling process. Altogether, by reducing the operation to two simple button presses and eliminating external power, the device achieves simplicity and affordability (∼USD 2), paving the way for widespread adoption of ISF‐based diagnostics in real‐world healthcare.

### 3.2 Characterization of the Applicator and Microneedle

To evaluate the structural integrity and functional performance, we characterized the applicator and microneedles through a series of experiments. Figure 2b shows photographs of the device from the front and back views, offering a direct visualization of its external appearance and complementing the schematic representation. Moreover, the mechanical properties during the artificial skin penetration process were also assessed, as shown in the force‐displacement curve (Figure 2c), where two distinct nodes (i and ii) corresponded to initial penetration and complete penetration to the skin, respectively. The reproducibility of these characteristic force values was further confirmed by three independent repeated experiments (Figure S8a,b). Figure 2d presents a pentagonal radar chart benchmarking system performance against reported representative works [6, 18, 19], representing the excellent performance in the five parameters: fabrication simplicity (enabled by one‐step 3D printing), speed (within 1 min), multiplex detection (simultaneous elemental and molecular analysis), operational simplicity (dual‐button design), and biosafety (minimally invasive sampling) (Figures S1–S3).

Then, the design and relevant properties of the microneedles were characterized. Figure 2e presents a schematic of the microneedles fabricated by SLA‐based 3D printing. The microneedles were designed with a 5 × 5 array, with a tip height of 1000 µm, inter‐needle spacing of 2100 µm, and base pore diameter of 600 µm (Figure S4). Figure 2f shows a photograph of a microneedle next to a 10 Singapore dollar coin, highlighting the miniature size of the designed microneedle. Two types of microneedle tip pores are compared in Figure 2g (i) and (ii). The oblique pore, used in this work, was fabricated by removing a small conical section from the base of the microneedle (Figure S6). This design offers two advantages over conventional straight pore: (1) preventing tissue clogging and (2) enhancing mechanical strength. Meanwhile, the force‐displacement curves were constructed in Figure 2g (iii) to evaluate the mechanical performance. Under the same force per needle, the displacement of the oblique pore was smaller than that of the conventional straight pore, indicating superior compressive mechanical performance. This difference was further validated by repeated measurements and statistical analysis of the force at 1 mm displacement (Figure S8c,d). To further evaluate its mechanical performance, the mechanical simulations of the tips were conducted by COMSOL (Figure S7). The results demonstrated that, under equivalent stress applied at the needle tips, the straight pore exhibited stress diffusion into the thin‐walled channel, increasing the risk of fracture. In contrast, the stress in the oblique pore was more concentrated in the thicker tip region, which exhibited greater mechanical strength.

In addition, the bottom and top views of the microneedles are shown in Figure 2h. These images illustrate the microneedle array and microfluidic channels. To evaluate the microneedles’ penetration capability, Figure 2i presents the insertion of a 2 × 2 microneedle array into an agarose‐based (2% (w/v)) artificial skin model. Additionally, the microneedle tips were coated with MB for subsequent fluorescence characterization. After penetration, the transdermal region of the skin model was observed using a fluorescence microscope, and both top‐view (Figure 2i (i)) and cross‐sectional images (Figure 2i (ii)) were captured. These observations confirmed the successful penetration of the microneedles into the artificial skin model. Figure 2j exhibits the SEM images of microneedle tips from different angles, revealing the structure of tips and holes to demonstrate the successful printing of microneedles.

**FIGURE 3:**
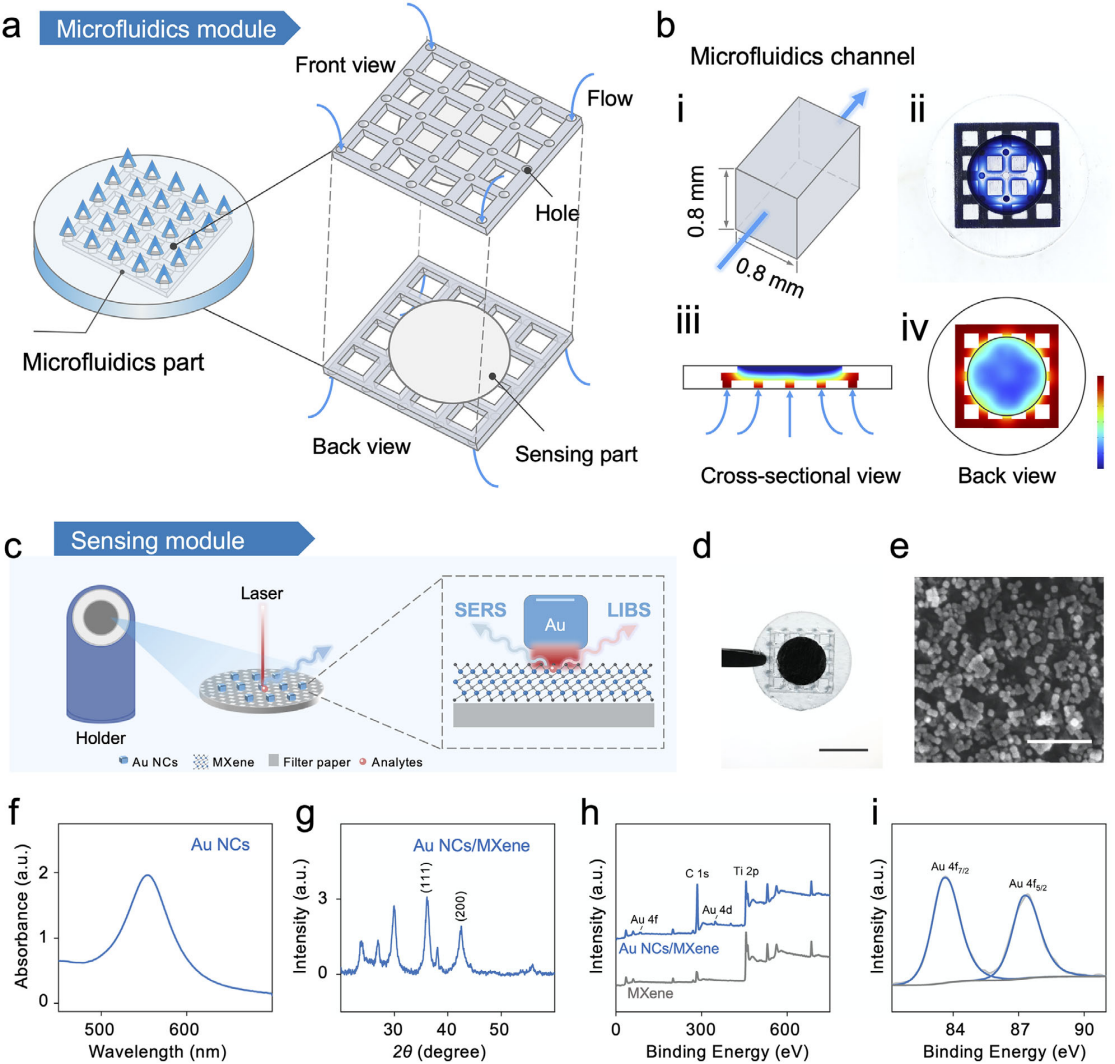
Characterization of the device's microfluidics and sensing modules. (a) Schematic showing the front view and back view of the microfluidics module. (b) (i). Schematic illustration of the microfluidics channel. (ii). Photography of microfluidic channel filled with MB dye. (iii, iv). Simulations of flow inside the channel with cross‐sectional and back views. (c) Schematic showing the work process of the sensing module. (d) Photography of the sensing module integrated with a microneedle with back view and front view. Scale bar: 1 cm. (e) SEM images of sensing module. Scale bar: 1 µm. (f) UV–vis spectrum of Au NCs. (g) XRD spectrum of Au NCs/MXene. (h) XPS spectra of MXene and Au NCs/MXene. (i) XPS spectrum of Au 4f region.

Following the characterization of the microneedle tips, the schematic of microfluidic channels, connected to the microneedle array, is demonstrated in Figure 3a. The front view schematic revealed the crisscrossing horizontal and vertical channels. Each intersection contained a pore, connecting with the through‐hole of the corresponding microneedle tip, enabling ISF entry into the microfluidics module. From the back view, the sensing module was located in the center of the microfluidic channels, allowing rapid and high‐throughput ISF collection. The size of the microfluidic channel interface is shown in Figure 3b (i), with a height and width of 0.8 mm. To validate the connectivity of the channel, the MB was introduced into the channels (Figure 3b (ii)). The successful diffusion of MB throughout the network validated the robust channel design. Additionally, COMSOL simulations were conducted to model the fluid dynamics within the microfluidic channels. The simulation results indicated that ISF enters through 25 interconnected pores at the microneedle‐microfluidic interface and gradually fills the entire channel network. Additionally, Figure 3b (iii) provides a cross‐sectional view, and Figure 3b (iv) illustrates the back view of the fluid distribution, highlighting the efficient flow and uniform filling of the microfluidic channels.

### 3.3 Preparation and Characterization of the Sensing Module

The sensing module for multi‐modal laser sensing was next characterized. Figure 3c illustrates the workflow of the sensing module. In detail, the module was excited to generate corresponding spectral data (SERS and LIBS), providing molecular and atomic information, respectively. The detailed preparation of the sensing module has been shown in Section 2.3. In this design, the MXene film loaded on filter paper serves as a conductive and adsorptive platform, while the uniformly distributed Au NCs act as plasmonic enhancers. Upon laser excitation, the strong LSPR of Au NCs significantly amplifies the electromagnetic field at the MXene surface, thereby boosting the Raman scattering signals of analytes adsorbed on the MXene substrate and enhancing the LIBS emission intensity. This synergistic effect enables simultaneous, high‐sensitivity SERS and LIBS detection. Figure 3d–i and Figures S11–S13 characterize the structural, optical, and chemical features of the Au NCs/MXene composite. Specifically, a photograph of the sensing module is presented in Figure 3d. The corresponding SEM image of the sensing module is shown in Figure 3 e, where the deposited Au NCs can be 、observed. Figure 3f shows the UV–vis absorption spectrum of Au NCs, featuring a pronounced plasmonic absorption peak centered at ∼550 nm. This peak originates from the localized surface plasmon resonance (LSPR) of the Au NCs and reflects their uniform morphology and narrow size distribution, both of which are critical for achieving reproducible and intense electromagnetic enhancement in SERS.

Figure 3g presents the XRD pattern of the Au NCs/MXene composite film. The diffraction peaks at 38.2° and 44.4° correspond to the (111) and (200) planes of face‐centered cubic Au. The coexistence of these peaks confirms the successful integration of Au NCs with MXene, and the retention of their distinct crystallographic features indicates that both components maintain excellent crystallinity after assembly—beneficial for preserving high plasmonic activity and electrical conductivity. Moreover, the XPS spectra of MXene and the Au NCs/MXene composite are exhibited in Figure 3h. In the composite, characteristic Au 4f peaks appear alongside the Ti 2p and C 1s peaks of MXene, confirming the presence of Au NCs on the MXene surface. Figure 3i presents the high‐resolution Au 4f XPS spectrum of the composite, showing two well‐defined peaks at 84.0 eV (Au 4f_7_/_2_) and 87.7 eV (Au 4f_5_/_2_), with no significant peak shift or broadening. This indicates that the Au NCs retain their metallic state and plasmonic properties after immobilization on MXene. Additional characterization results are provided in the (Figures S11–S13). Collectively, these results demonstrate that the sensing module possesses excellent plasmonic activity, providing a robust foundation for high‐sensitivity and reproducible multimodal SERS–LIBS detection.

### 3.4 In Vitro Characterization

**FIGURE 4:**
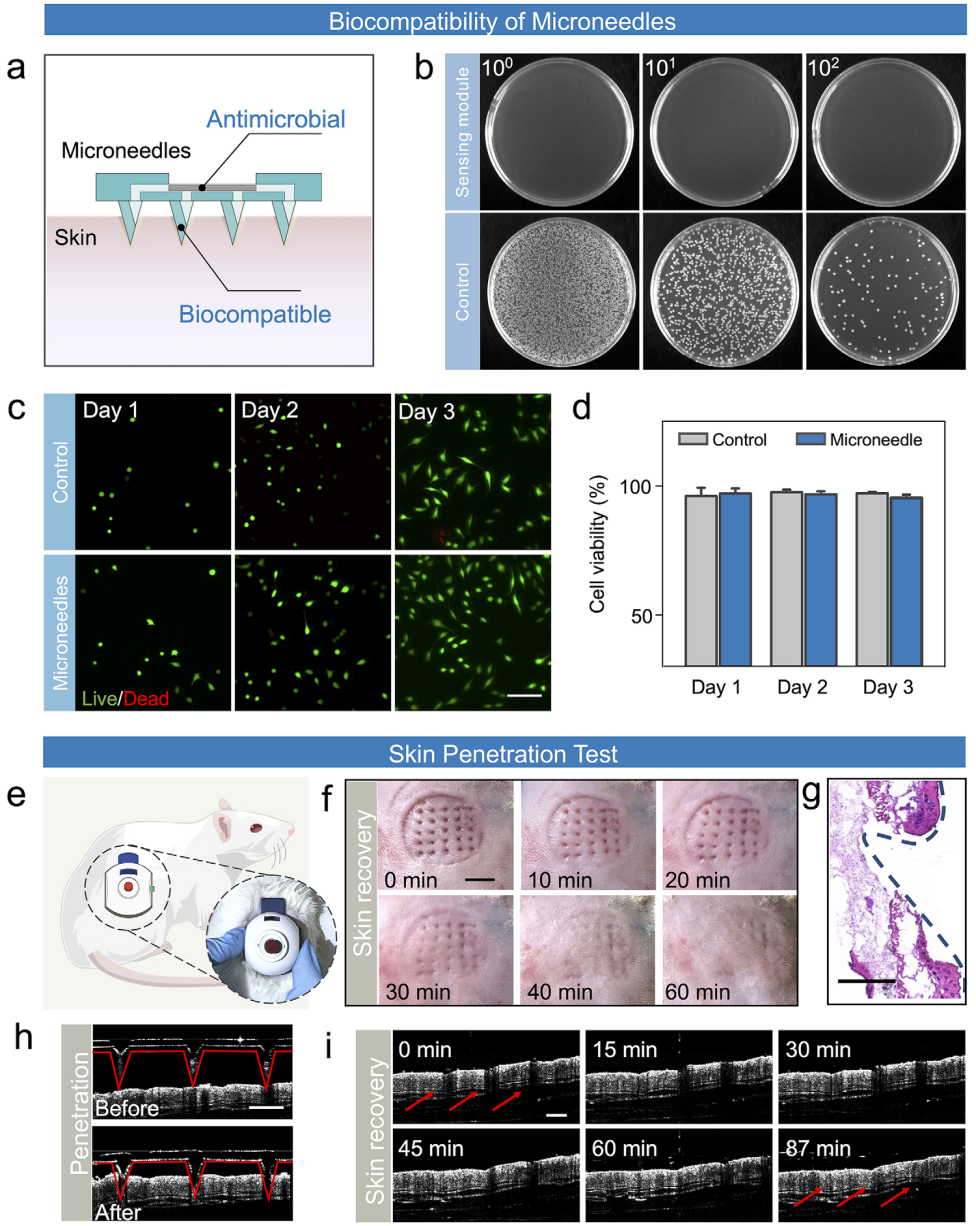
Characterization of microneedles’ biocompatibility and skin penetration ability. (a) Schematic showing the microneedles with an antimicrobial sensing module and biocompatible tips. (b) Photography of the antibacterial test on the sensing module and the control group. (c) Live/dead cell staining images for biocompatibility testing of the microneedle group compared with the control group, with a bar chart (d) of cell viability. Scale bar: 100 µm. (e) Schematic illustration of skin penetration test on a rat's back with photography of the operation. (f) Photographs of skin recovery after skin penetration. Scale bar: 500 µm. (g) Microscopic image of skin staining after skin penetration. Scale bar: 500 µm. (h) OCT images show the microneedles’ penetration process. Scale bar: 1 cm. (i) OCT images showing skin recovery process. Scale bar: 500 µm.

A series of in vitro experiments was conducted to assess the biocompatibility and performance of the device. Figure 4a shows the testing requirements for each part of the microneedle and sensing components. Each of the components underwent specific evaluations to ensure safety and functionality. For example, the sensing module was tested for its antibacterial activity, which is important for long‐term use in clinical settings. In addition, a biocompatible test was performed on the microneedle tips, which directly contact the skin. The antibacterial performance of the sensing module is presented in Figure 4b and Figure S14, including four experimental groups. No obvious bacterial colonies were observed on the sensing module, whereas clear colony growth was found on the bare filter paper, demonstrating the strong antibacterial activity of the sensing module. In addition, the MXene‐only filter paper showed negligible colony formation, while the Au nanocubes‐only filter paper exhibited clear bacterial colonies, indicating that MXene is the dominant contributor to the antibacterial performance of the sensing module. Figure 4c,d exhibit the in vitro biocompatibility results, including live/dead cell staining images for both the test and control groups over days 1, 2, and 3. The images show that the number of live cells increased gradually in both groups, with no significant differences observed. Quantitative analysis of cell growth was conducted to provide more details about the microneedle system's biocompatibility.

Subsequently, the transdermal performance of the microneedles was evaluated using healthy adult SD rats (Figure 4e). After shaving the dorsal fur, the microneedles were applied to penetrate the skin for 1 min, and the skin was observed for any potential injury. Figure 4f shows the skin recovery process following microneedle penetration. At 0 min, puncture marks appeared on the skin. Over time, the marks gradually faded and were nearly undetectable by 60 min, demonstrating rapid healing after microneedle penetration. Figure 4g presents H&E staining results of the puncture sites, demonstrating that the microneedle penetrated the epidermis to a depth of about 600 µm.

To further examine transdermal properties, OCT was employed for real‐time monitoring (Figure 4h,i; Figure S9). Figure 4h exhibits the penetration process, showing the microneedle tip contacting the skin and then inserting into the tissue. Figure 4i records skin recovery after microneedle removal. At 0 min, three puncture marks were visible. As time passed, the marks faded, becoming hard to detect at 60 min and disappearing by 87 min, showing the skin's healing ability. These results demonstrated that the microneedles penetrate the skin with minimal residual damage. The rapid healing process supports their biocompatibility, making them suitable for biomedical applications.

### 3.5 Validation of Multi‐Modal Laser Sensing

**FIGURE 5:**
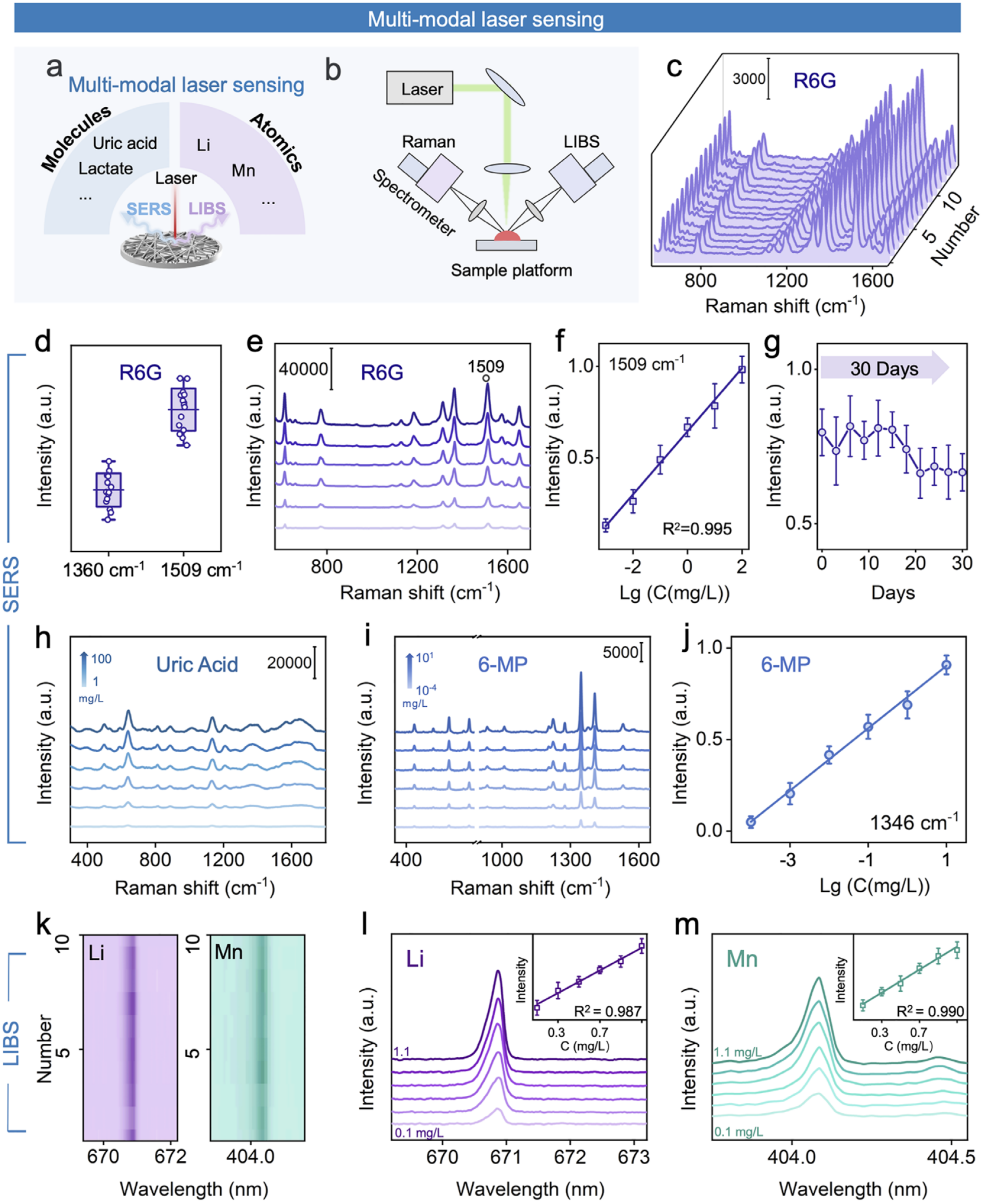
Characterization of multi‐modal laser sensing. (a) Schematic showing multi‐modal laser sensing to obtain molecular and atomic information. (b) Schematic illustration of a simplified spectroscopic setup. (c) Waterfall plot of 15 SERS spectra for R6G at 10^−3 ^mg/L, with Box plot (d) of SERS peak intensity at 1360 and 1509 cm^−1^. (e) SERS spectra of R6G with 6 gradient concentrations from 10^−3^ to 10^2^ mg/L, with a calibration curve of peak intensity at 1509 cm^−1^. (g) Line chart showing peak intensity at 1509 cm^−1^ change within 30 days. SERS spectra with gradient concentration for uric acid (h) and 6‐MP (i). (j) The calibration curve of 6‐MP at 1346 cm^−1^ with gradient concentration. (k) Waterfall plot of LIBS spectra for Li and Mn. (l) LIBS spectra with gradient concentration for Li at 670.87 nm with the related calibration curve. (m) LIBS spectra with gradient concentration for Mn at 404.08 nm with the related calibration curve.

Figure 5 illustrates the comprehensive characterization of the sensing module for multi‐modal laser‐sensing. Figure 5a presents a schematic of the multi‐modal spectral sensing system, which acquires both SERS and LIBS spectra to further obtain molecular and atomic information, respectively. Specifically, SERS can detect molecular biomarkers from ISF such as glucose, uric acid, and urea, while LIBS can identify atomic biomarkers, including Li, Na, Mn, etc. Figure 5b exhibits the schematic diagram of the spectroscopic setup. The system consists of a laser for sample excitation and CCD‐equipped spectrometers to capture the emitted light and convert it into corresponding spectral data.

First, to evaluate the SERS performance of the sensing module, the molecular R6G was selected. A droplet (2 µL, 10^−1^ mg/L) of R6G solution was deposited onto the sensing module, dried at 60°C, and subjected to SERS analysis. Fifteen spectra were collected and plotted as a waterfall diagram in Figure 5c, qualitatively revealing the consistency and stability (RSD = 9.4%) of the sensing module for SERS detection. For quantitative analysis, the intensities of the 15 R6G spectra at characteristic peaks (1509 and 1360 cm^−^^1^) were recorded and displayed as box plots (Figure 5d). The RSD of spectral intensities was 3.7% at 1509 and 4.9% at 1360 cm^−^^1^, demonstrating the promising stability and reproducibility of the sensing module for SERS detection.

Subsequently, the qualification and quantification performance of SERS were conducted on R6G solutions with gradient concentrations in Figure 5e. For analytes with a wide dynamic range (e.g., R6G), the gradient concentrations were prepared on a logarithmic scale (10‐fold intervals) to assess linearity over multiple orders of magnitude (e.g., 10^−3^, 10^−2^, 10^−1^, 10^0^, 10^1^, and 10^2^ mg/L). The results indicated the increase in spectral intensity with rising of R6G concentrations. Then, a calibration curve was established by plotting the intensity at 1509 cm^−^^1^ against the concentration (Figure 5f). For each concentration, 20 spectra were collected and averaged. The calibration curve (R^2^ = 0.995) demonstrated high precision and quantitative capability of the sensing module. To evaluate the shelf life of the sensing module, daily measurements were conducted over 30 days, with 20 spectra collected and averaged each day. The results, plotted in a line graph (Figure 5g), show an overall signal decay of 16.2% over 30 days, indicating a satisfactory shelf life and operational stability.

To further validate the sensing module's applicability for physiologically relevant molecules, additional experiments were conducted on typical molecule biomarkers (uric acid and 6‐MP). In contrast to R6G, these analytes were tested using linear (equal‐interval) concentration gradients to mimic realistic physiological fluctuations. Specifically, the gradient concentration of uric acid (Figures 5h, 1, 20, 40, 60, 80, and 100 mg/L) was analyzed by SERS. The results demonstrated that the spectral intensities of the characteristic peaks increased with the increase in biomarker concentrations. Moreover, 6‐MP with gradient concentration (10^−4^, 10^−3^, 10^−2^, 10^−1^, 10^0^, and 10^1 ^mg/L) was analyzed (Figure 5i) for subsequent in vivo pharmacokinetic studies and the calibration curve at 1346 cm^−1^ was constructed in Figure 5j (R^2^ = 0.997), exhibiting good linearity. These results further demonstrated the sensing module's precision and universal applicability for biomarker detection.

Meanwhile, the LIBS performance of the sensing module was also evaluated. First, LIBS spectra of common elements (Na, K, and Ca) in the human body were analyzed to verify the primary detection capability of the sensing module (Figures S15–S19). Then, to evaluate the sensitivity of the sensing module, trace metal elements commonly found in biological systems (Li and Mn) were selected as representative targets. Specifically, a 2 µL droplet of Li or Mn solution at a concentration of 1 mg/L was deposited onto the sensing module and analyzed by LIBS. Ten spectra were collected for each sample and plotted as top‐view waterfall diagrams (Figure 5k). The average intensities and RSD of Li (4586, 13.7%) and Mn (3027, 9.4%) indicate consistent signal reproducibility. Subsequently, LIBS detection of gradient concentrations of Li (0.1, 0.3, 0.5, 0.7, 0.9, and 1.1 mg/L) was performed in Figure 5l, where the Li peaks’ (670.87 nm) intensities increased with the increase of Li concentration. And the calibration curve of Li at 670.87 nm demonstrated good linearity (R^2^ = 0.987). Moreover, the spectral intensities (Figure 5m) of Mn (404.08 nm) increased with the increase of Mn concentration (0.1, 0.3, 0.5, 0.7, 0.9, and 1.1 mg/L). The calibration curve based on the peaks exhibited promising linearity with R^2^ = 0.990. In summary, the LIBS characterization further confirmed the sensing module's capability of elemental analysis. Together, these results establish the multi‐modal sensing module as a robust and versatile platform for biomolecular and elemental analysis.

### 3.6 Animal Pharmacokinetic Experiment and AI‐Driven Diagnosis

To validate the feasibility of the device, an animal pharmacokinetic study was conducted, with experimental procedures detailed in Section 2.8. ISF samples were analyzed for 6‐MP using SERS, and for Li using LIBS. Standard techniques, LC‐MS and ICP‐MS, were employed to detect 6‐MP and Li in plasma, and the results were compared with those obtained from SERS and LIBS.

**FIGURE 6:**
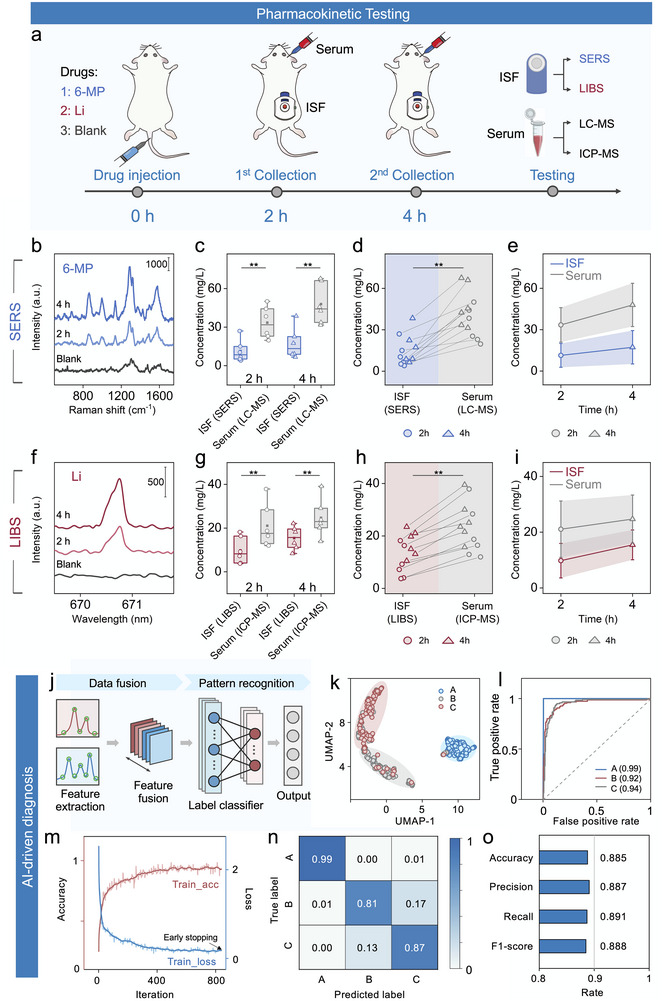
Characterization of pharmacokinetic testing and AI‐driven diagnosis. (a) Schematic illustration of pharmacokinetic testing, including drug injection, collection, and testing. (b) SERS spectral comparison (blank, 2 collection, 4 h collection) of 6‐MP on the sensing module. (c) Box plots showing comparative analysis of 6‐MP concentrations in ISF (SERS) and serum (LC‐MS) across 2 an 4 h collection. (*p* < 0.05). (d) Scatterplot illustrates the correlation between 6‐MP concentrations measured in ISF (SERS) and serum (LC‐MS) across 2 an 4 h collection. (e) Line plots showing the change of 6‐MP concentration in ISF and serum, respectively. (f) LIBS spectral comparison (blank, 2 collection, 4 h collection) of Li on the sensing module. (g) Box plots showing comparative analysis of Li concentrations in ISF (LIBS) and serum (ICP‐MS) across 2 an 4 h collection. (*p* < 0.05). (h) Scatterplot illustrates the correlation between Li concentrations measured in ISF (LIBS) and serum (ICP‐MS) across 2 an 4 h collection. (i) Line plots showing the change of Li concentration in ISF and serum, respectively. (j) Schematic of an AI‐driven diagnosis framework. (k) UMAP visualization of CNN‐extracted spectral features. (l) ROC curves for multi‐class classification using the fused spectral inputs. (m) Learning curves of the CNN‐based model during training and validation. (n) Confusion matrix of classification results from the CNN‐based model. (o) Bar chart summarizing model evaluation metrics: accuracy, precision, recall, and F1‐score.

The SERS analysis of 6‐MP is shown in Figure 6b–e. First, Figure 6b compares the SERS spectra of ISF samples collected at 2 and 4 h from group A and at 2 h from group C. Characteristic peaks of 6‐MP were visible in both 2 and 4 h ISF samples, indicating that the microneedle device collects and detects 6‐MP in ISF. The 4 h sample showed higher 6‐MP signal intensity than the 2 h sample, demonstrating that 6‐MP diffuses from plasma into ISF within the first 0–4 h post‐injection.

Figure 6c presents box plots comparing the 6‐MP concentrations in ISF (detected by SERS) at 2 and 4 h from Group A with plasma concentrations (detected by LC‐MS) at the same time points. The results showed that the 6‐MP concentration in ISF (mean concentration) was lower than that in plasma (mean concentration) (^**^ *p* < 0.05), though both were within the same order of magnitude, indicating that 6‐MP injected into the bloodstream had not fully diffused into ISF. Figure 6d provides scatter plots showing the distribution of 6‐MP concentrations in ISF and plasma at 2 and 4 h, with concentrations in ISF lower than in plasma (^**^ *p* < 0.05).

Furthermore, Figure 6e exhibits the time‐dependent changes in 6‐MP concentrations in plasma and ISF at 2 and 4 h post‐injection. The 6‐MP concentration in plasma increased from 33.5 to 47.9 mg/L (43.0% increase), while the ISF concentration rose from 11.4 to 17.3 mg/L (51.8% increase). These findings indicated that 6‐MP continues to diffuse from plasma into ISF after intravenous injection, reflecting the absorption and distribution of the drug.

Then, LIBS was used for the qualitative and quantitative analysis of Li. Plasma samples were analyzed using ICP‐MS as a reference. Figure 6f shows the LIBS spectra of ISF samples collected from group B at 2 and 4 h, compared to the blank control spectra from group C. Characteristic peaks for lithium (670.8 nm) were observed in both the 2 and 4 h ISF samples, indicating that the microneedle device collected and detected Li in ISF. Moreover, the 4 h sample showed higher Li signal intensity than the 2 h sample, reflecting a higher Li concentration at 4 h. Figure 6g presents box plots comparing the Li concentrations in ISF (detected by LIBS) at 2 and 4 h from Group A with plasma concentrations (detected by ICP‐MS) at the same time points. The results showed that the Li concentration in ISF was lower than in plasma. Figure 6h provides scatter plots showing the distribution of Li concentrations in ISF and plasma, with concentrations in ISF lower than in plasma (^**^ *p* < 0.05). Figure 6i shows the changes in Li concentrations in plasma and ISF at 2 and 4 h post‐injection. The Li concentration in plasma increased from 21.1 to 24.7 mg/L (17.1% increase), while the ISF concentration increased from 9.8 to 15.5 mg/L (58.2% increase).

These experimental trials validated that the device, integrated with multimodal laser sensing, enables the detection of small‐molecule drugs and trace elements in ISF with good sensitivity and time resolution. The concentrations of 6‐MP and Li in ISF increased over time, following similar trends to those in plasma, confirming that ISF can serve as a minimally invasive alternative for drug monitoring. Although ISF concentrations were lower than plasma levels, their temporal trends were aligned. The larger relative increases in ISF (e.g., 51.8% for 6‐MP and 58.2% for Li) indicate the system's ability to track drug distribution over time.

### 3.7 AI‐Driven Data Processing for Diagnosis

As part of the device workflow, we implemented a companion AI‐driven module to transform SERS and LIBS spectra into diagnostic readouts delivered to users. To enable efficient interpretation of complex multimodal spectral data, this AI‐driven module was established to perform automated feature extraction, data fusion, and classification. Figure 6j exhibits the workflow of the AI‐driven diagnostic model, which employs a CNN‐based spectral fusion architecture. Specifically, SERS and LIBS spectra, collected from the sensing module, were first processed for feature extraction and fusion. The fused features were then subjected to pattern recognition, enabling accurate classification and diagnosis. (Details of machine learning algorithms were provided in Figures S20 and S21). Figure 6k shows the 2D visualization of the CNN‐extracted fused spectral features using UMAP. The three groups (A, B, and C) formed distinct clusters in the 2D space. Notably, the group A was well separated from the B and C groups, while B and C exhibited closer proximity but still maintained distinguishable distributions. These results indicated that the CNN‐based spectral fusion architecture effectively captures discriminative features among the different time points. To further assess classification performance, the ROC curves (Figure 6i) were built with AUC values to further assess the performance of the deep learning model. The AUC values for the 3 groups were all greater than 0.92, demonstrating high accuracy of classification. Figure 6m indicates the training dynamics of the CNN model, describing the train process. After training, the train loss curve converged to 0 gradually, while the train accuracy curve converged to 1. These learning curves confirm that the model was well‐trained and reached convergence without overfitting.

The confusion matrix for the CNN‐based model is shown in Figure 6n, the 2 H group was identified with 99% accuracy. Moreover, misclassifications between the B (17%) and C (13%) groups were observed, due to both groups receiving drug injections, resulting in similar spectral profiles. The bar chat in Figure 6o displayed the classification evaluation indicators of the CNN‐based model. The results of the three indicators (Accuracy: 0.885, precision rate: 0.887, recall rate: 0.891, and F1‐score: 0.888) demonstrated excellent classification performance. These results demonstrated the robustness and reliability of the AI‐driven diagnostic model for multimodal spectral classification and diagnosis.

## 4 Conclusion

We presented a portable, user‐friendly, and dual‐button device that enables rapid ISF sampling and multimodal laser sensing for POCT applications. The integrated dual‐button applicator facilitates controlled skin insertion and removal, minimizing user error and contamination risks. The 3D‐printed porous microneedles with slanted microfluidic channels ensure efficient ISF sampling within 1 min. By combining SERS and LIBS, the device enables simultaneous detection of molecules and electrolytes in ISF. Moreover, the AI‐driven data processing further enhances the interpretation of complex spectral data, supporting reliable classification and diagnostic decisions (All accuracy > 0.88). Both in vitro and in vivo studies confirm the device's performance in tracking clinically relevant analytes with high accuracy and stability (All R^2^ > 0.98). Overall, this study presents a practical strategy that integrates minimally invasive sampling devices with laser sensing technologies, providing a scalable and translational pathway toward decentralized healthcare and precise disease monitoring.

## Author Contributions

**Shengqun Shi**: investigation, visualization. **Lianbo Guo**: writing – review and editing, supervision. **Xiujuan Hu**: investigation, formal analysis. **Yunchen Long**: validation, writing – review and editing. **Yuanchao Liu**: writing – original draft, conceptualization, writing – review and editing. **Bowen Li**: writing – review and editing, validation. **Chaochao Sun**: investigation, visualization. **Annan Chen**: investigation, visualization, writing – review and editing. **Condon Lau**: writing – review and editing, supervision, funding acquisition. **Bingbing Gao**: investigation, formal analysis. **Chwee Teck Lim**: visualization, writing – review and editing. **Zhixing Ge**: validation, visualization. **Wei Luo**: writing – review and editing, validation.

## Conflicts of Interest

The authors declare no conflicts of interest.

## Supporting information




**Supporting File**: advs75564‐sup‐0001‐SuppMat.docx.

## Data Availability

The data that support the findings of this study are available from the corresponding author upon reasonable request.
