# Supplementary material for: A Portable and Dual‐Button Microneedle Device Enables Intelligent Multimodal Laser Sensing
Source: Adv Sci (Weinh). 2026 May 7;13(43):e75564. doi: 10.1002/advs.75564 (PMC13336013; doi:10.1002/advs.75564)
Supplement: Supplementary file 1 — Supporting File: advs75564‐sup‐0001‐SuppMat.docx. [file ADVS-13-e75564-s001.docx]

**Supporting Information:**

**A portable and dual-button microneedle device enables intelligent multimodal laser sensing**

Yuanchao Liu^1^, Xiujuan Hu^1^, Shengqun Shi^8^, Bowen Li^6^, Zhixing Ge^2^, Yunchen Long^6^, Chaochao Sun^5^, Annan Chen^5^, Bingbing Gao^7^, Lianbo Guo^4*^, Condon Lau^1*^, Wei Luo^3*^, Chwee Teck Lim^2,9*^

1. *Department of Physics, City University of Hong Kong, Kowloon 999077, Hong Kong SAR, China*
2. *Department of Biomedical Engineering, National University of Singapore, Singapore 117583, Singapore*
3. *School of Integrated Circuits, Huazhong University of Science and Technology, Wuhan 430074, China*
4. *Wuhan National Laboratory for Optoelectronics (WNLO), Huazhong University of Science and Technology, Wuhan 430074, China*
5. *Department of Mechanical Engineering, City University of Hong Kong, Kowloon, Hong Kong SAR 999077, China*
6. *Department of Materials Science and Engineering, City University of Hong Kong, Kowloon, Hong Kong SAR 999077, China*
7. *School of Pharmaceutical Sciences, Nanjing Tech University, Nanjing 211816, China*
8. *College of information and Intelligence Engineering, Zhejiang Wanli University, Zhejiang 315000, China*
9. *Institute for Health Innovation and Technology, National University of Singapore, Singapore 117599, Singapore*

** Corresponding authors: Condon Lau (condon.lau@cityu.edu.hk), Lianbo Guo (lbguo@hust.edu.cn), Wei Luo (luowei@hust.edu.cn), Chwee Teck Lim (ctlim@nus.edu.sg)*

This section includes the following:

1. Figure S1-S22


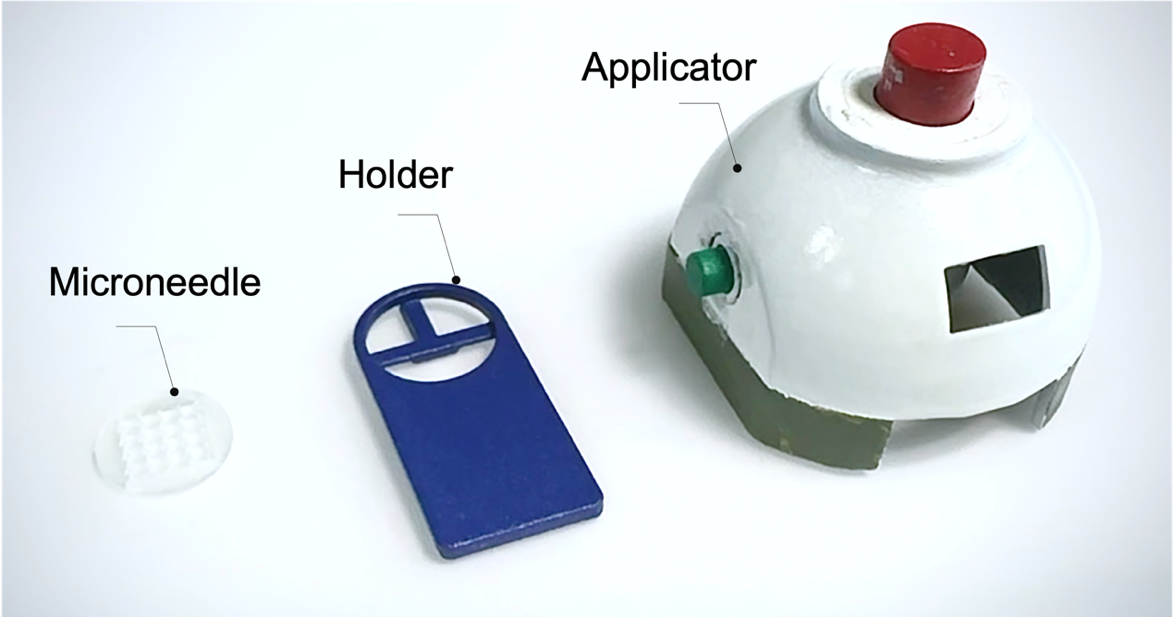


**Fig. S1.** Photographs of the components of the device, including the microneedle, holder, and applicator.


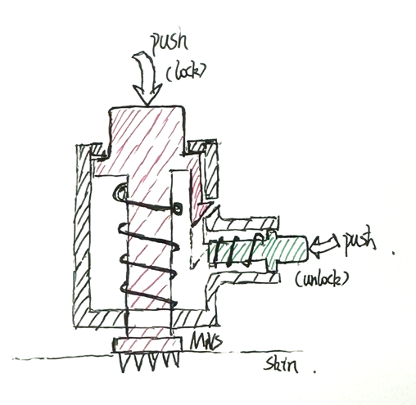


**Fig. S2.** A hand-drawn schematic illustrating the internal structure and working principle of the device.


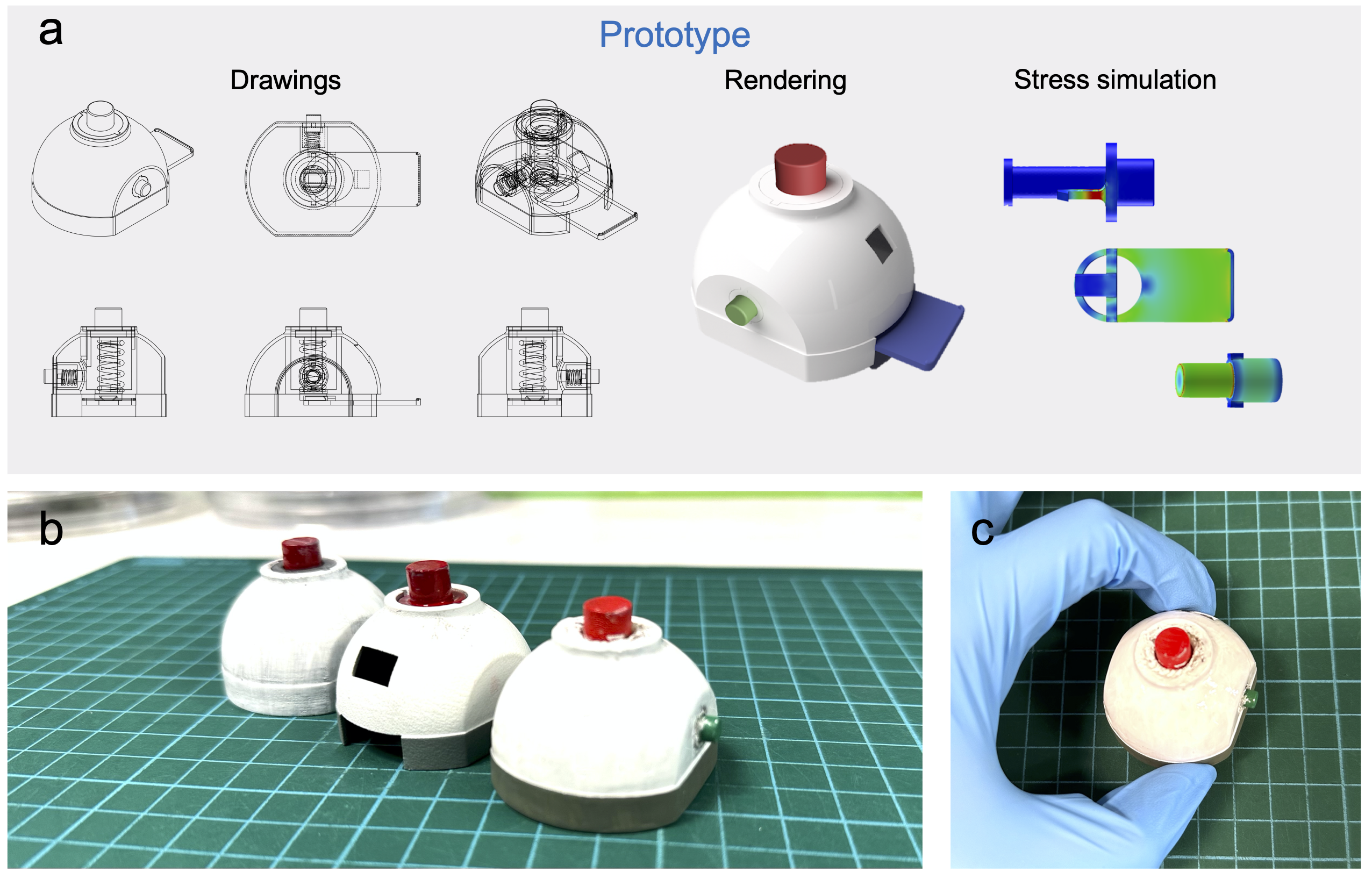


**Fig. S3.** (a) Perspective views of the device prototype from multiple angles, rendered images, and stress simulations of selected components. (b) and (c) Photographs of semi-finished components coated with color.


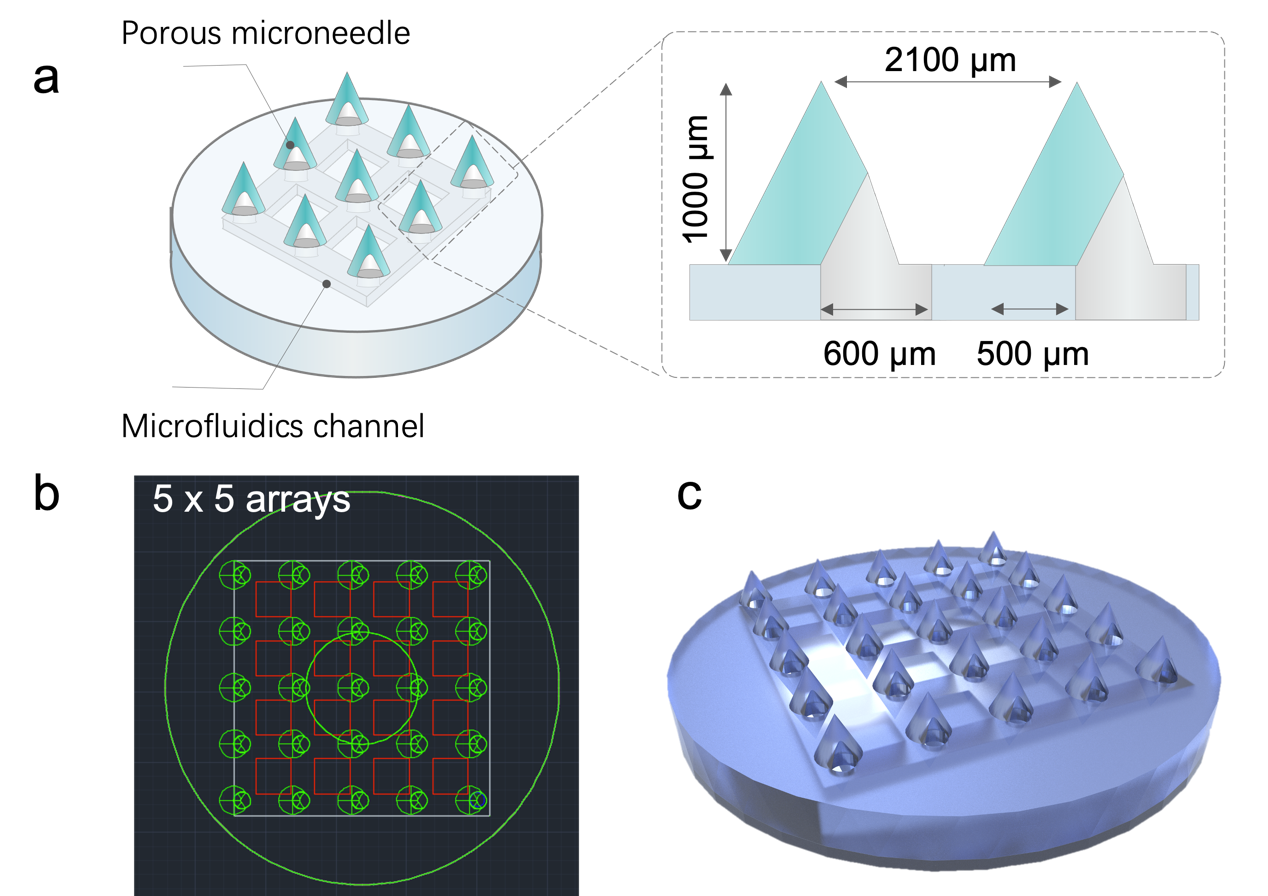


**Fig. S4.** (a) Schematic design and dimensions of the microneedle structure, (b) line drawing and (c) rendered image.


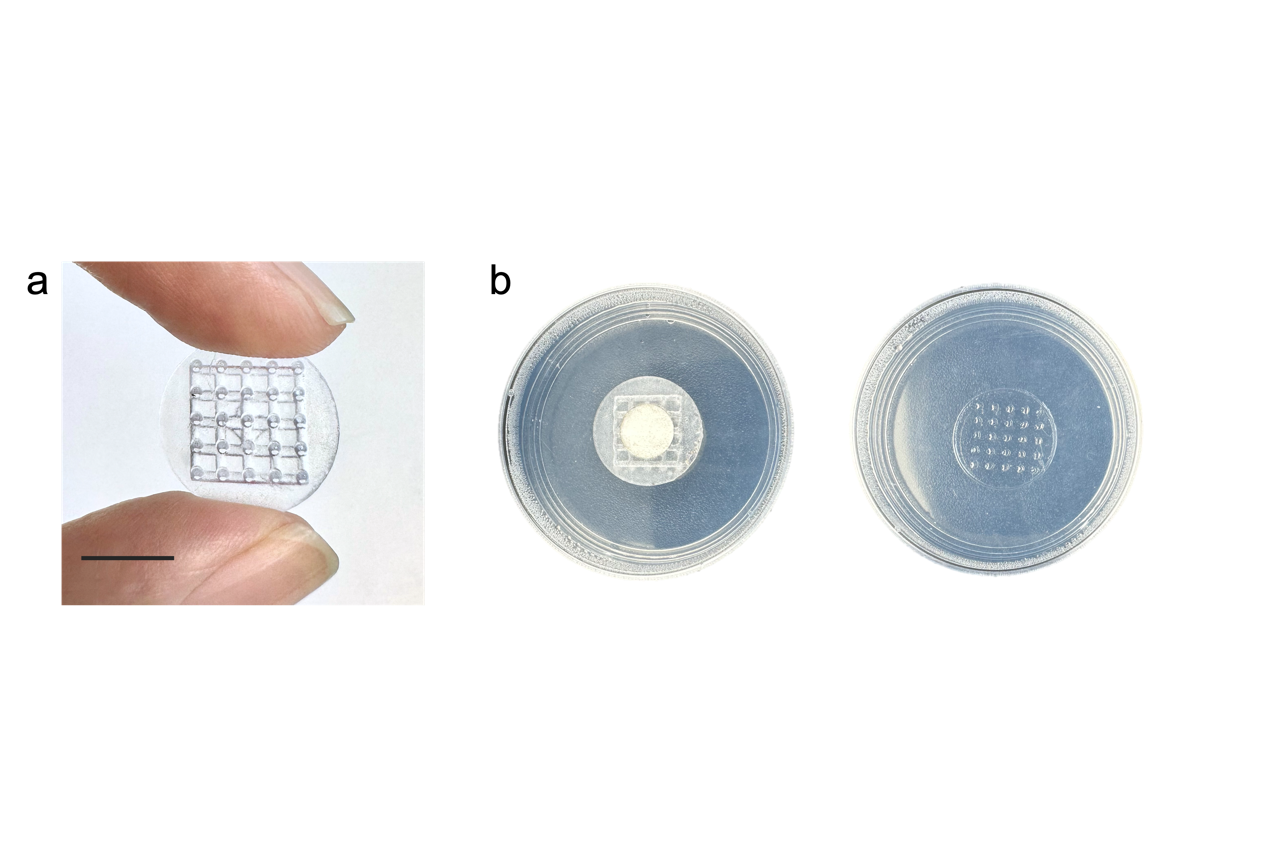


**Fig. S5.** (a) Macro photography of the microneedle structure. (b) Microneedle penetration into the PDMS substrate, leaving traces.


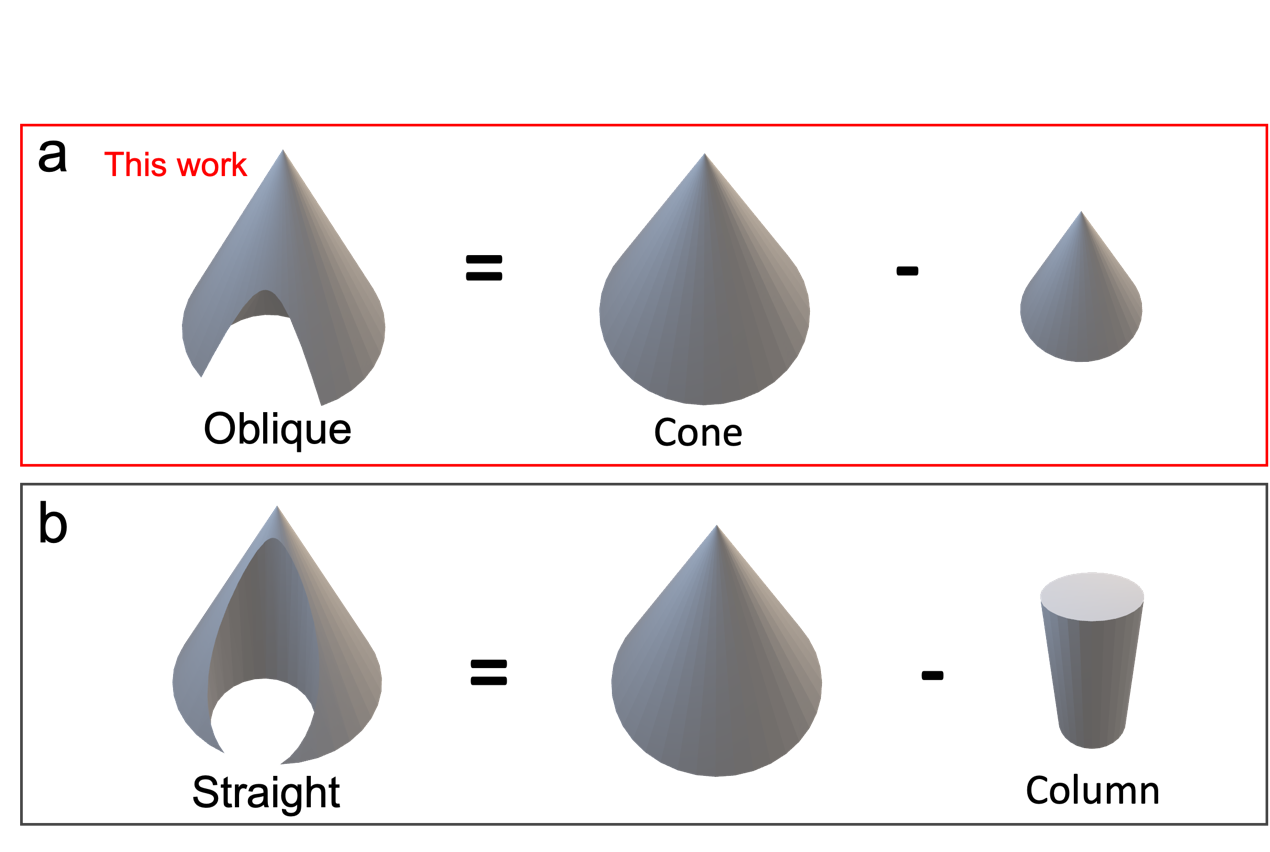


**Fig. S6.** (a) Designed microneedle tip with oblique channel in this work. (b) Microneedle tip with straight channel.


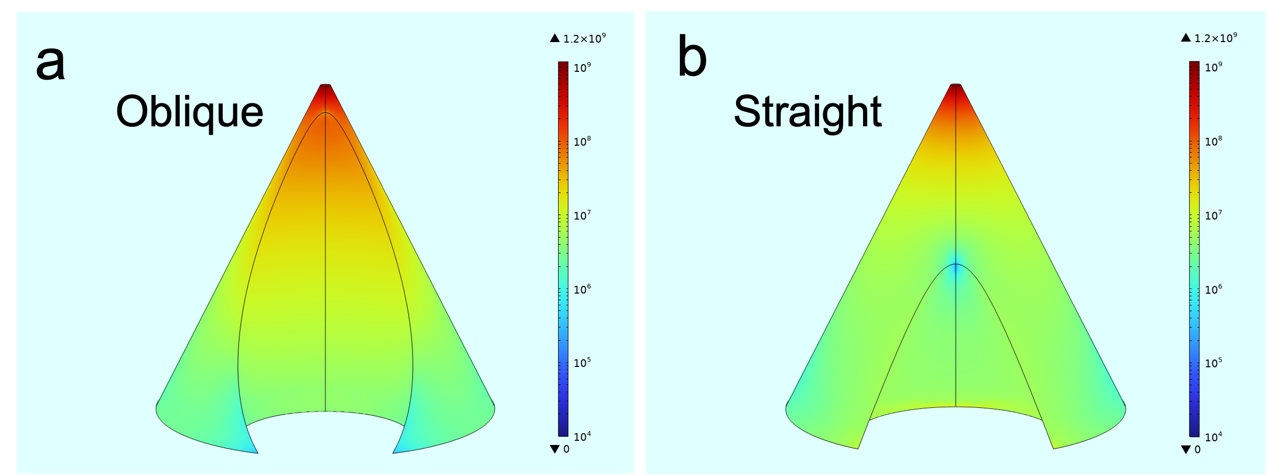


**Fig. S7.** Finite element simulation of the mechanical stress distribution in needle tips with (a) an oblique channel and (b) a straight channel. The color map indicates the stress magnitude.


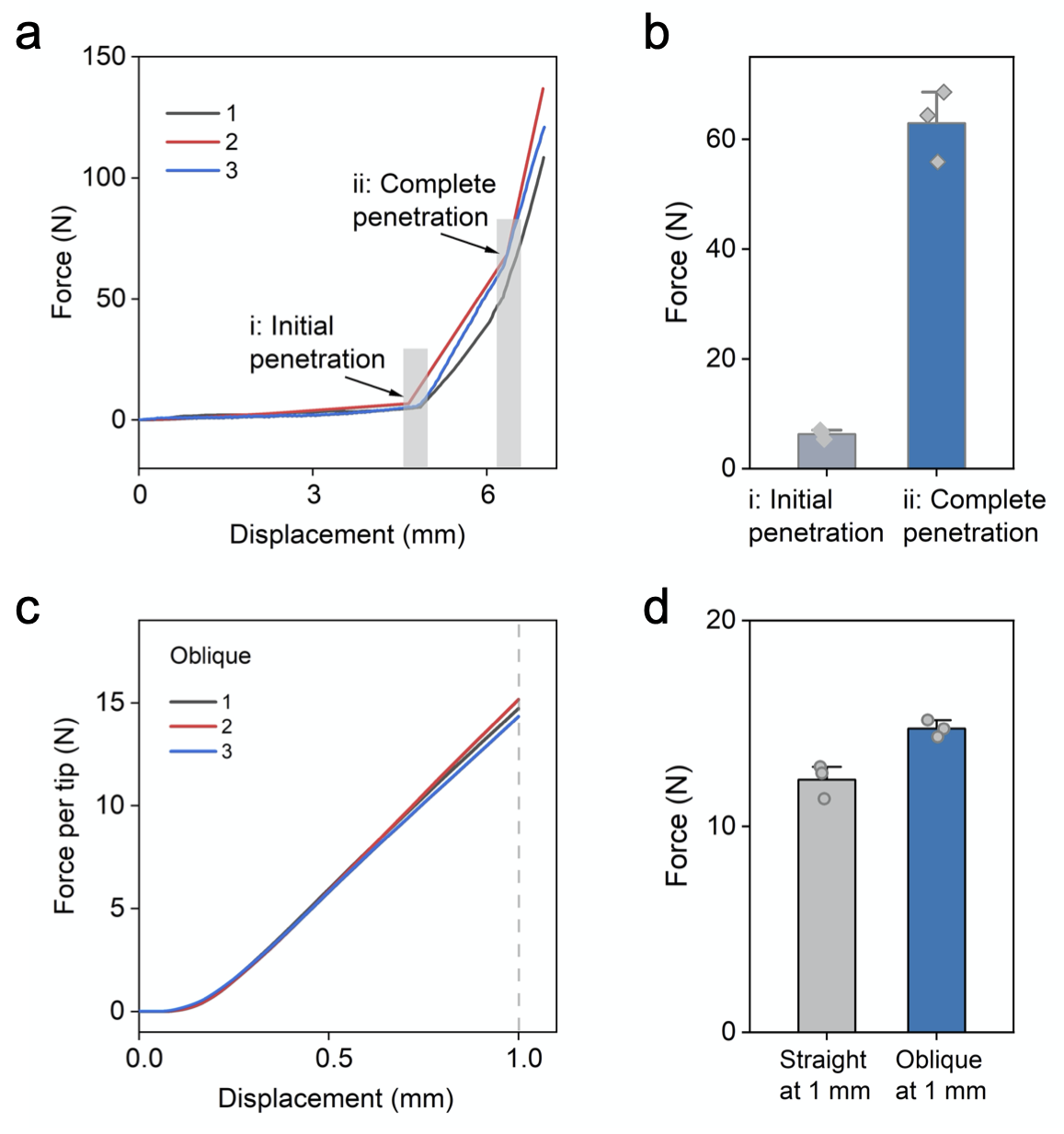


**Fig. S8** (a) Three repeated force–displacement curves of the device during penetration into artificial skin. (b) Statistical analysis of the characteristic forces corresponding to initial penetration and complete insertion (n = 3). (c) Three repeated force–displacement curves for microneedle tips with oblique channels. (d) Statistical comparison of the force at 1 mm displacement for straight and oblique channel designs (n = 3).


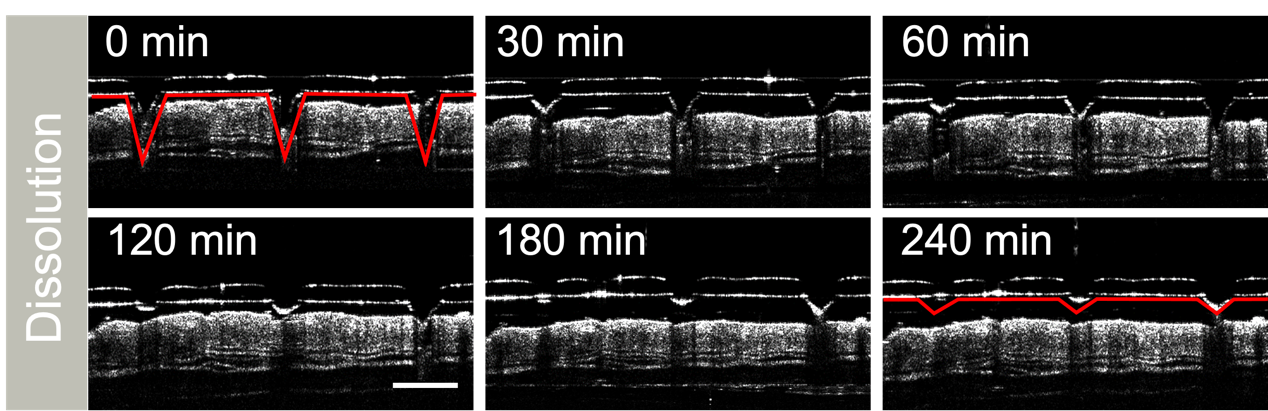


**Fig. S9.** OCT images recording the process of microneedle insertion into the skin and its dissolution after 240 min.


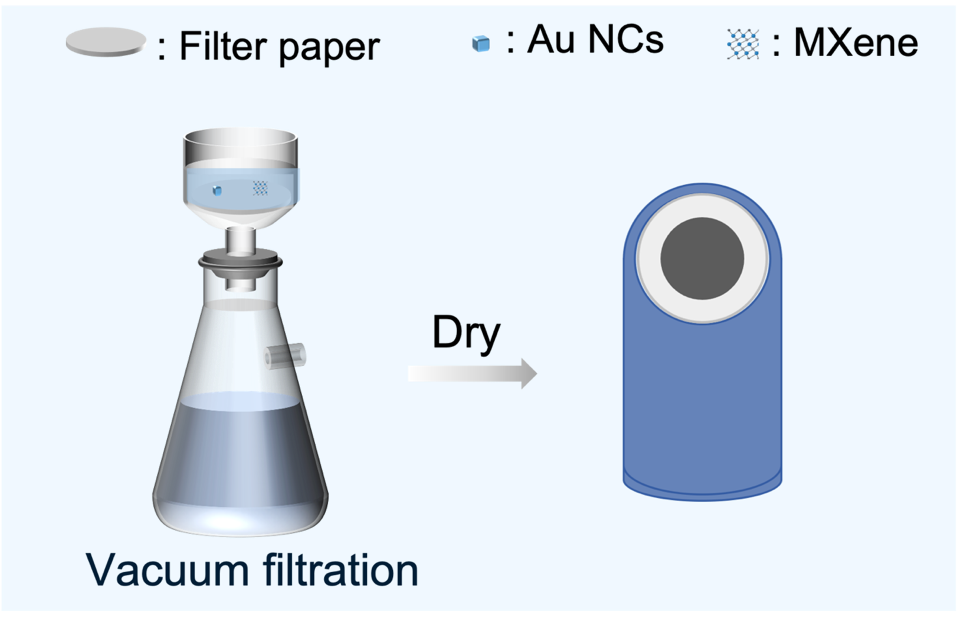


**Fig. S10.** Schematic illustration of the fabrication process of sensing module.


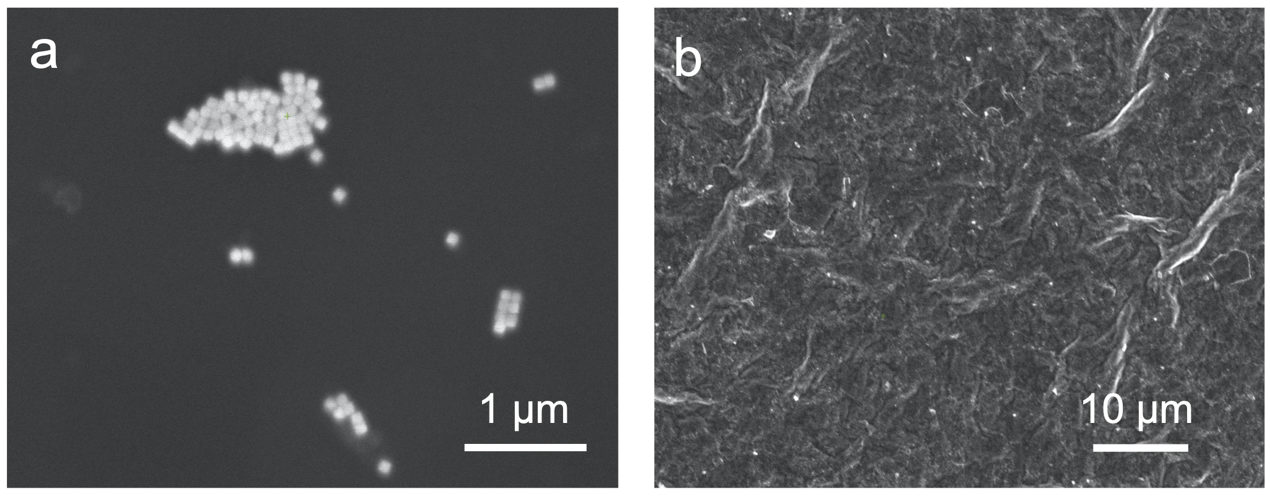


**Fig. S11.** SEM image of (a) Au NCs and (b) MXene.


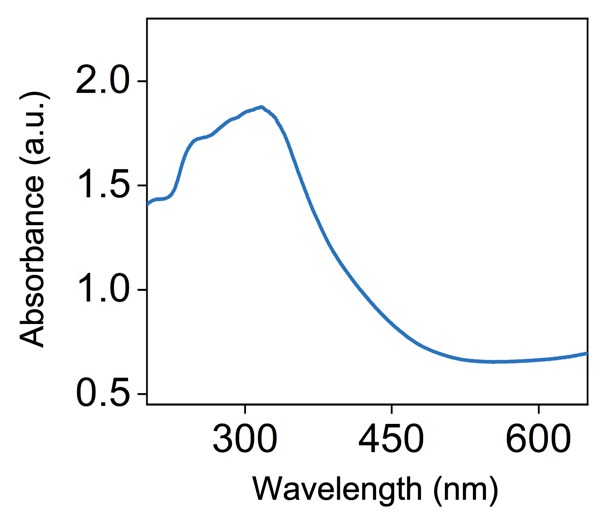


**Fig. S12.** UV-vis spectrum of MXene.


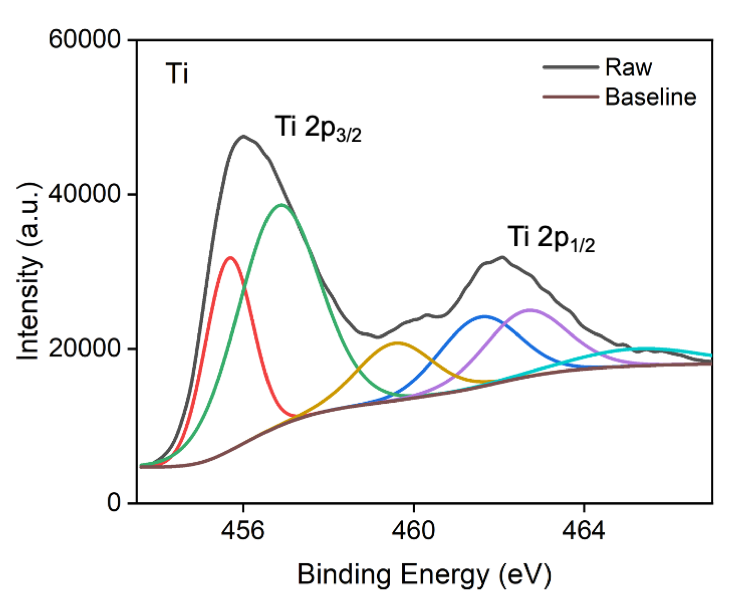


**Fig. S13.** High-resolution XPS spectrum of Ti 2p. The black line shows the raw experimental spectrum, the brown line denotes the fitted baseline, and the colored lines represent the deconvoluted sub-peaks assigned to Ti 2p₃/₂ and Ti 2p₁/₂.


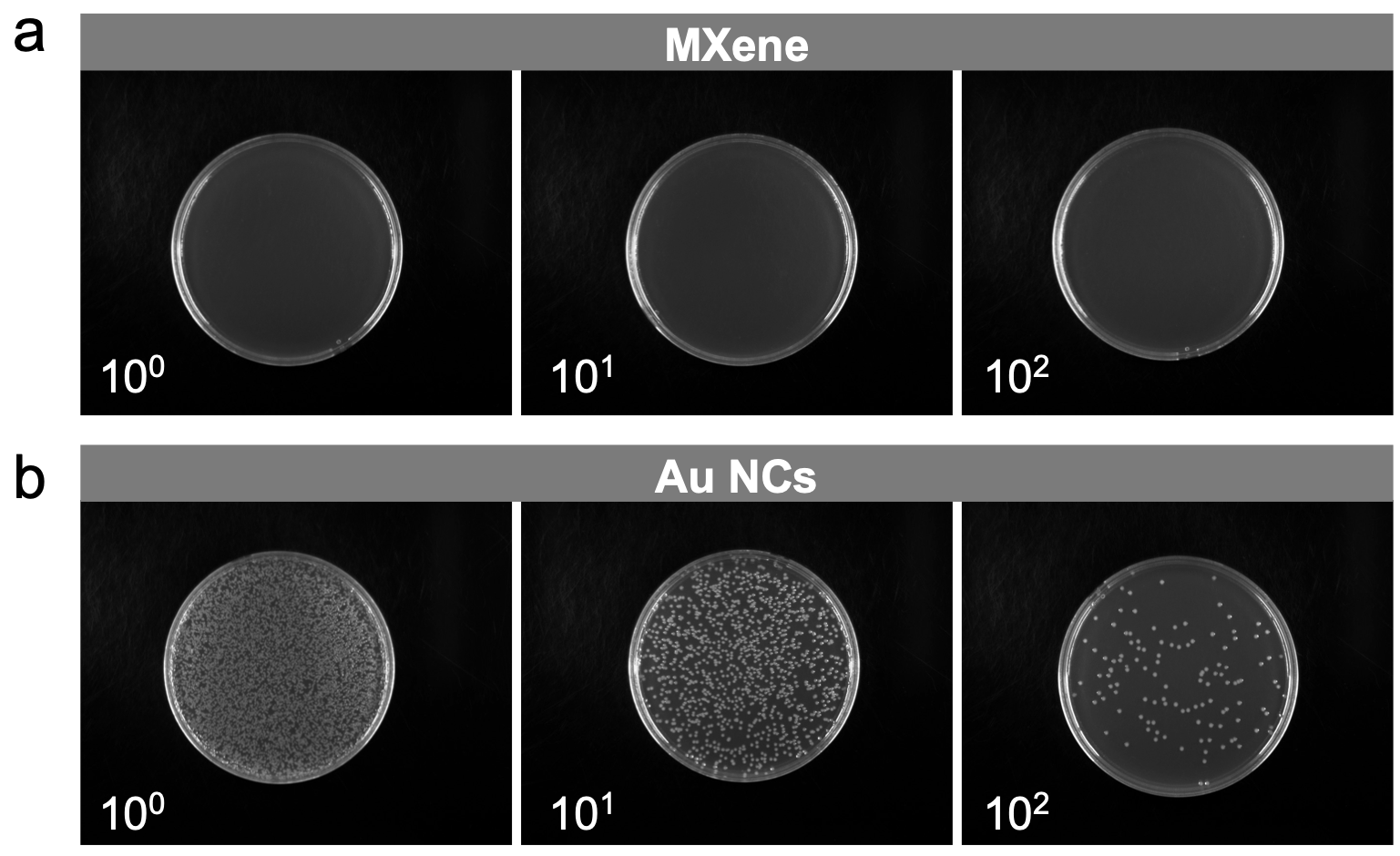


**Fig. S14.** Photography of antibacterial test on the (a) MXene-only filter paper and (b) Au NCs only-filter paper group.


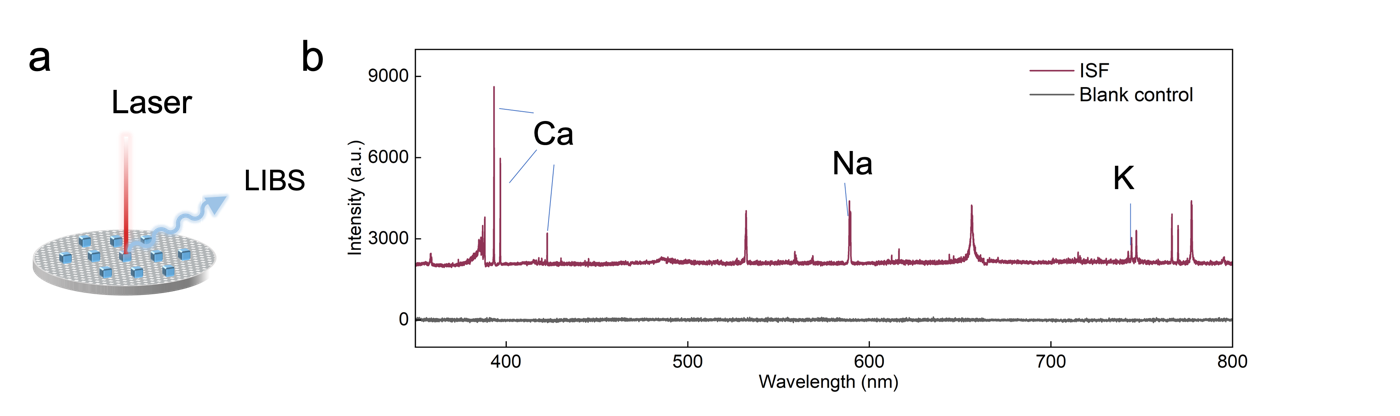


**Fig. S15.** (a) Schematic illustration of LIBS spectral acquisition. (b) Full-range LIBS spectra of ISF applied onto the sensing module, with blank filter paper substrate used as the control.


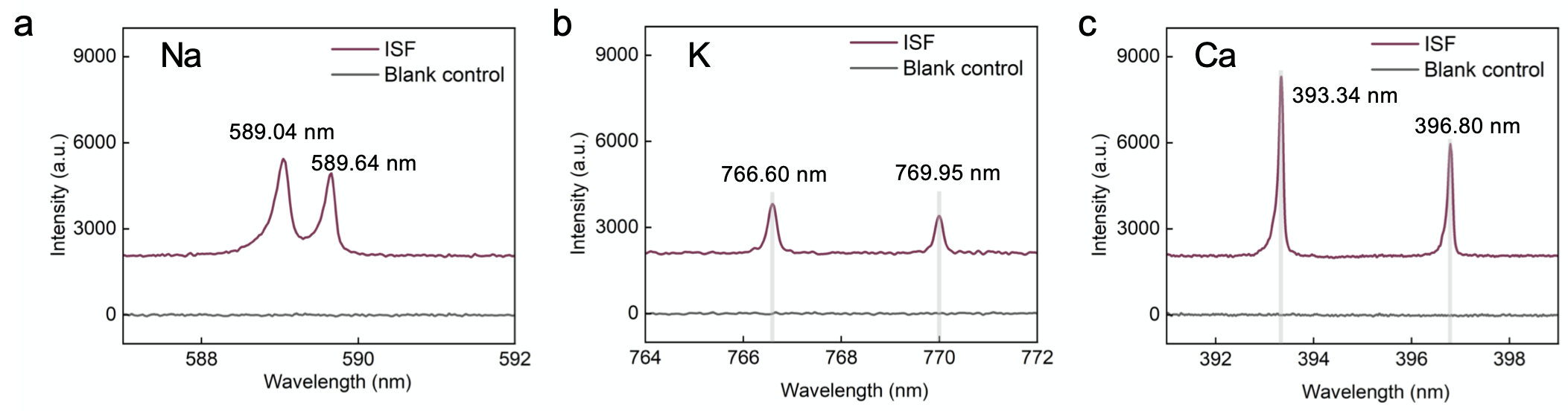


**Fig. S16.** Characteristic LIBS spectral lines of Na (a), K (b), and Ca (c) element.


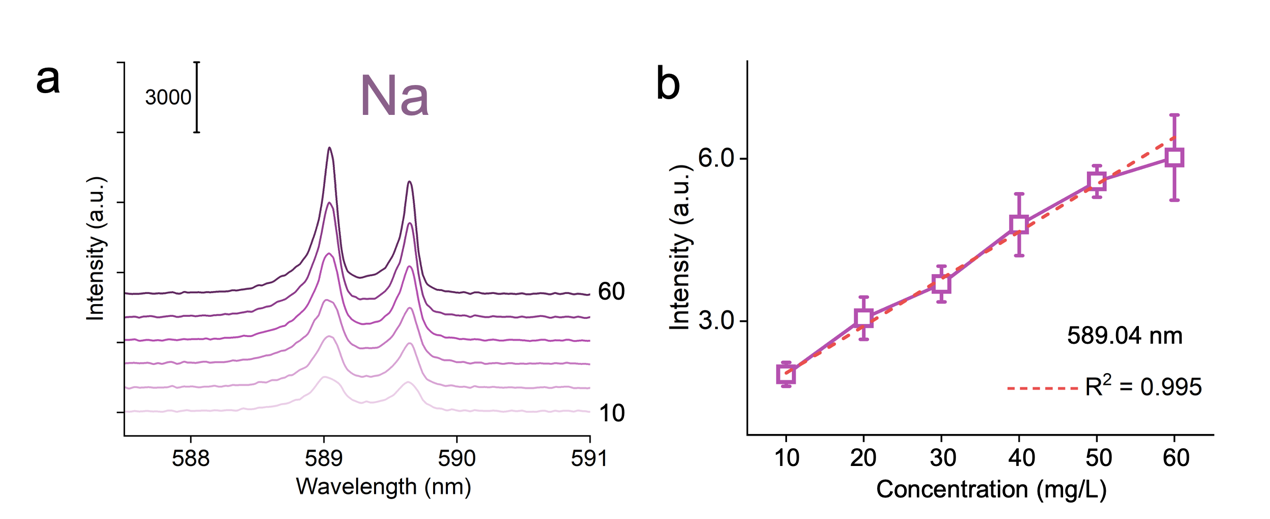


**Fig. S17.** (a) LIBS spectra of Na at varying concentrations; (b) Corresponding calibration curve of Na, illustrating the relationship between LIBS signal intensity and concentration.


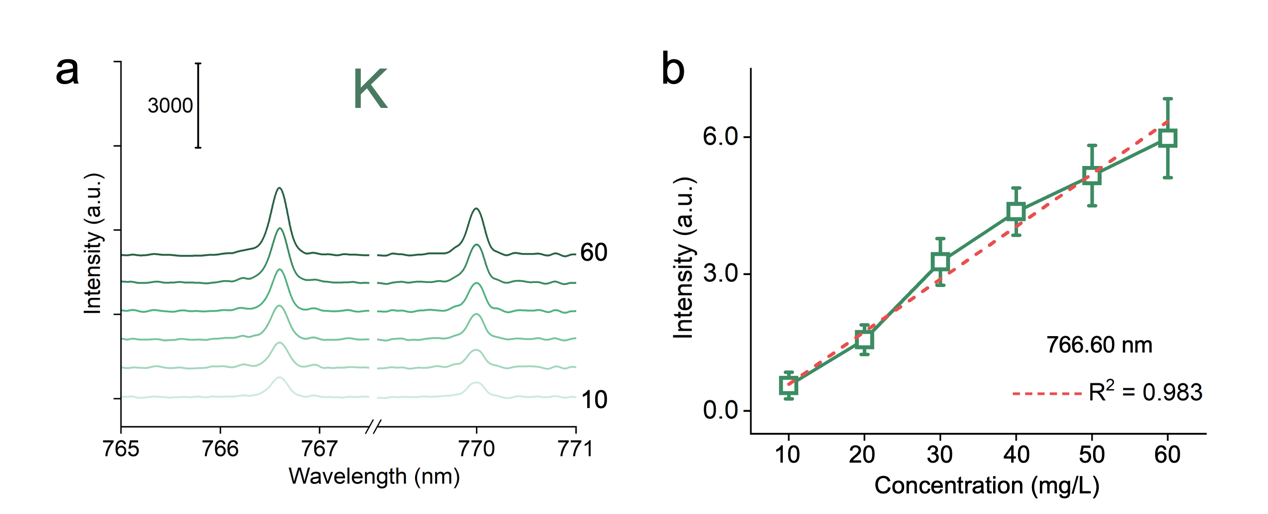
**Fig. S18.** (a) LIBS spectra of K at varying concentrations; (b) Corresponding calibration curve of K, illustrating the relationship between LIBS signal intensity and concentration.


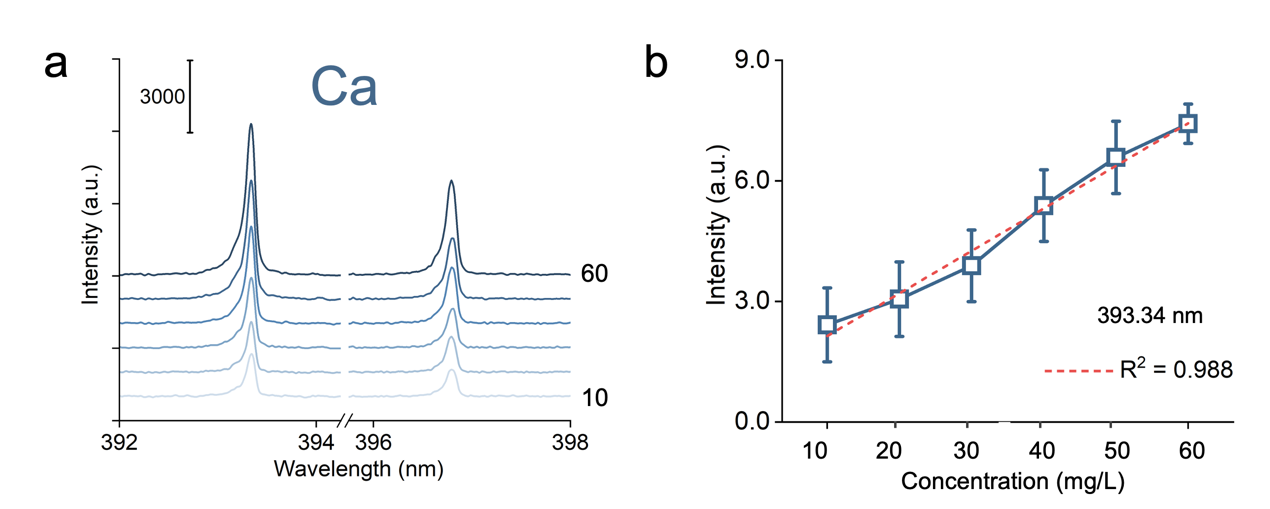


**Fig. S19.** (a) LIBS spectra of Ca at varying concentrations; (b) Corresponding calibration curve of Ca, illustrating the relationship between LIBS signal intensity and concentration.


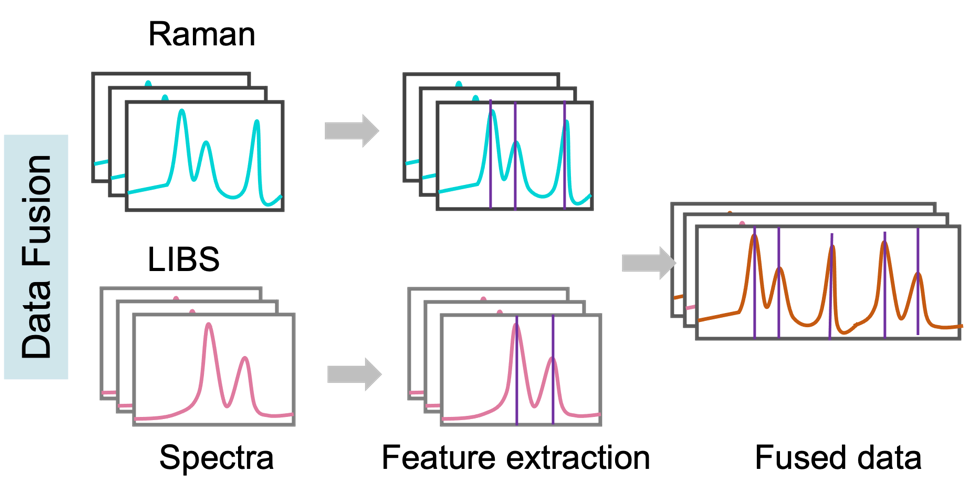


**Fig. S20.** Schematic illustration of data fusion based on Raman and LIBS spectral information.


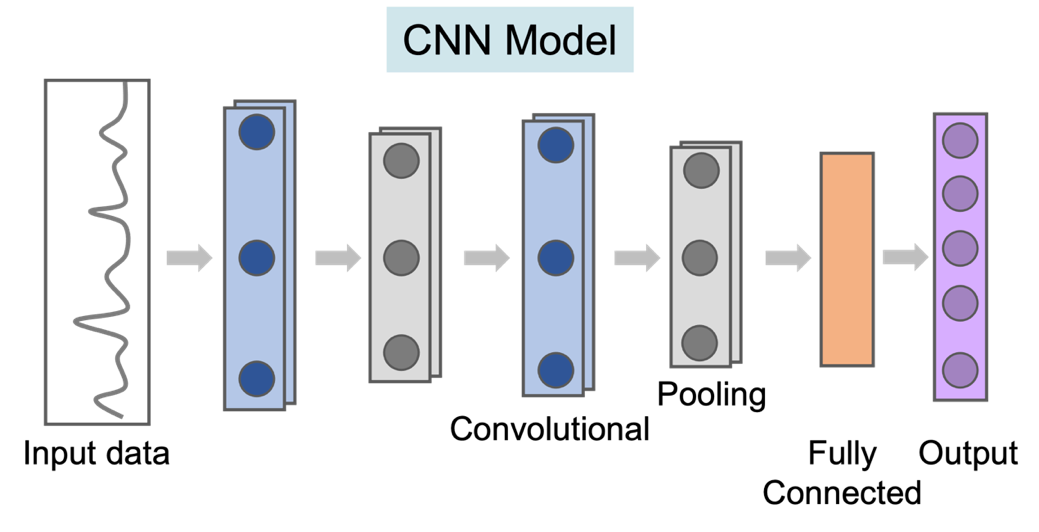


**Fig. S21.** Schematic illustration of the CNN model used in this work.


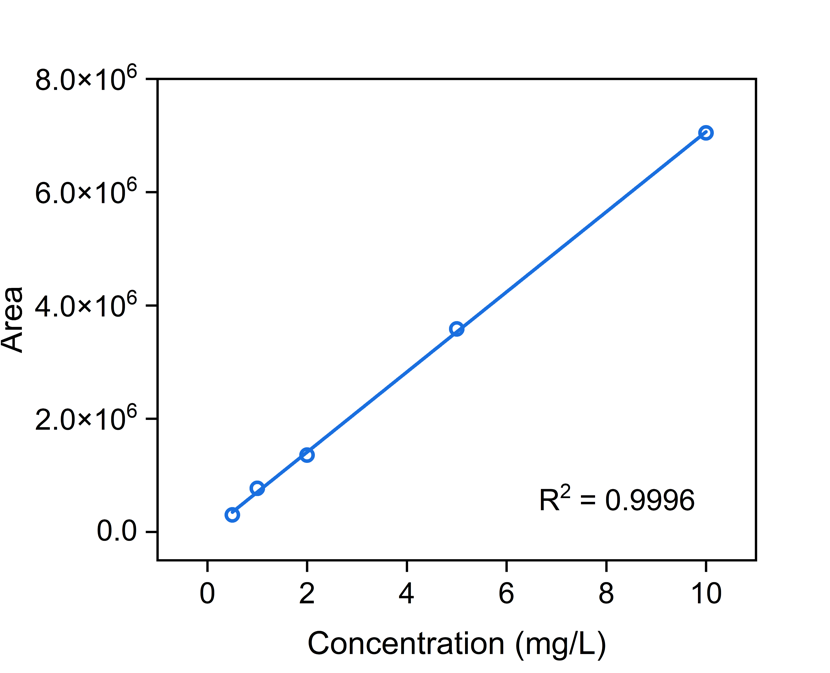


**Fig. S22.** LC-MS calibration curve for 6-MP, showing the relationship between peak area and concentration.
